# Resolving intra-tumor heterogeneity and clonal evolution of core-binding factor acute myeloid leukemia patients with single-cell resolution

**DOI:** 10.1186/s40164-025-00718-4

**Published:** 2025-10-28

**Authors:** Raphael Hablesreiter, Paulina M. Strzelecka, Klara Kopp, Natalia Estrada, Anna Dolnik, Marlon Tilgner, Coral Fustero-Torre, Felicitas Thol, Florian H. Heidel, Michael Heuser, Laleh Haghverdi, Lars Bullinger, Friederike Christen, Frederik Damm

**Affiliations:** 1 Corporate Member of Freie Universität Berlin and Humboldt-Universität Zu Berlin, Department of Hematology, Oncology and Cancer Immunology, Charité – Universitätsmedizin Berlin, Augustenburger Platz 1, 13353 Berlin, Germany; 2 Department of Hematology, Hemostasis, Oncology and Stem Cell Transplantation, Hannover Medical School (MHH), Hannover, Germany; 3 Leibniz Institute on Aging, Fritz-Lipmann-Institute, Jena, Germany; 4 Department of Internal Medicine IV, University Hospital Halle (Saale), Martin-Luther-University Halle-Wittenberg, Halle, Germany; 5 Max-Delbrück-Center for Molecular Medicine in the Helmholtz Association (MDC), Berlin Institute for Medical Systems Biology (BIMSB), Berlin, Germany; 6 German Cancer Consortium (DKTK), Partner Site Berlin and German Cancer Research Center (DKFZ), Heidelberg, Germany

**Keywords:** AML, CBF, Intra-tumor heterogeneity, Clonal evolution, Clonal heterogeneity, Single-cell DNA sequencing

## Abstract

Reconstructing and understanding intra-tumor heterogeneity, the coexistence of multiple genetically distinct subclones within the tumor of a patient, and tumor development is essential for resolving carcinogenesis and for identifying mechanisms of therapy resistance. While bulk sequencing can provide a broad view on tumoral complexity/heterogeneity of a patient, single-cell analysis remains essential to identify rare subclones that might drive chemotherapy resistance. In this study, we performed an integrated analysis of bulk and single-cell DNA sequencing data of core-binding factor acute myeloid leukemia patients, defined by the presence of a *RUNX1::RUNX1T1* or *CBFB::MYH11* fusion gene. By single-cell sequencing, we inferred tumor phylogenies for 8 patients at diagnosis including patient-specific somatic variants, somatic copy-number alterations and fusion genes, and studied clonal evolution under the pressure of chemotherapy for 3 patients. As a result, we developed an approach to reliably integrate subclonal somatic copy number alterations into phylogenetic trees and clonal evolution analysis, obtaining unprecedented resolution of intra-tumor heterogeneity in CBF AML. We were able to show that the fusion gene is among the earliest events of leukemogenesis at single-cell level. We identified remaining tumor clones in 6 patients with complete remission samples indicating incomplete eradication of the tumor clones. Here, we show that identifying the order of mutation acquisition can provide valuable insights into evolutionary history, offering a framework to improve drug selection in the era of targeted therapies.

**Supplementary Information:**

The online version contains supplementary material available at 10.1186/s40164-025-00718-4.

To the editor,

Intra-tumor heterogeneity (ITH) describes the coexistence of multiple genetically distinct subclones within the tumor of a patient resulting from somatic evolution, clonal diversification and selection [1]. Core-binding factor (CBF) acute myeloid leukemia (AML) is characterized by the presence of a translocation of chromosomes (chr) 8 and 21 [t(8;21)(q22;q22)] or an inversion/translocation of chr 16 [inv(16)(p13.1q22) or t(16;16)(p13.1q22)] resulting in *RUNX1::RUNX1T1* and *CBFB::MYH11* fusions, respectively [2]. We and others resolved the mutational composition and evolutionary patterns of CBF AML with conventional next-generation sequencing techniques [3–5]. Although modelling clonal trajectories from bulk sequencing has provided important insights, single-cell resolution is necessary to unravel true clonal composition and evolution.

We developed an approach for the systematic integration of single-cell DNA (scDNA-seq) and bulk sequencing to unravel ITH and subclonal architecture. We analyzed samples from 2 patients with t(8;21) and 7 with inv(16) (Tables S1,S2) by whole exome (WES), targeted and nanopore sequencing (diagnosis [D]:9, complete remission [CR]:7, relapse [Rel]:8 samples) as well as targeted scDNA-seq (D:9, CR:7, Rel:5 samples). Sample/material availability and sequencing status is detailed in Table S3 and the Material and Method section.

We identified 405 variants via bulk sequencing as previously described (Table S4) [6, 7]. 232 (mean = 25.8) and 173 (mean = 21.6) variants in diagnosis (n = 9) and relapse (n = 8) samples were detected, respectively (Figs. S1-S4). Additionally, we identified 7 somatic copy-number alterations (SCNAs) via WES and defined CBF fusion gene breakpoints by nanopore sequencing (Fig. S5 and Tables S5, S6). By using custom panels covering patient-specific somatic variants, SCNAs and CBF fusions (Table S7), a median of 4103 cells/sample were sequenced (range:711–7560) with a mean coverage of 106 reads/amplicon/cell (range:35–384, Figs. S6-S9, Tables S8, S9) [8] and a high concordance between bulk and scDNA-seq variants (Figs. S10-S12). The median allele dropout (ADO) rate in the samples ranged from 12.9%-21.8% with individual ADO rates per amplicon from 0.9%-27.1% (Fig. S9).

A 2-step approach for assigning copy-number profiles to inferred tumor phylogenies from COMPASS [9] was developed, which allowed identification of subclonal SCNAs that were not supported by single nucleotide variants (SNVs) and missed using existing computational methods [9, 10]. We inferred tumor phylogenies for 8 patients at diagnosis (Figs. S13, S14). Phylogenetic trees were constructed using reference and alternative counts, without incorporating genotype or zygosity information to account for observed variety in read depth, allelic imbalance and ADO rates of investigated amplicons. Patient 03 was excluded from phylogenetic analysis due to low variant overlap between bulk and scDNA-seq (Fig. S10) and the lack of inv(16) detection on single-cell level (Fig. S15). We identified 3–11 (mean = 5.6) AML clones per patient. The *CBFB::MYH11* fusion was part of the founding clone in the remaining 6 patients with inv(16). *RUNX1::RUNX1T1* was acquired early in both patients with t(8;21). Interestingly, a low number of cells (patient 01:14 at D, 44 at D and Rel combined; patient 09: 39 at D) acquired mutations before the t(8;21) translocation (Fig. S16-S21) which is in concordance with the higher rate of co-mutations in patients with t(8,21) [11]. Those earlier clones harbored mutations in genes that are not known AML driver mutations (*ZBTB17*, *ARV1*, *SCN1B*, *CYP8B1*, *PHIP*, *EIF2B4*, *LAMB4*, *NWD1*). As a result, leukemogenesis was likely initiated by the *RUNX1::RUNX1T1* fusion. We detected a higher fraction of mutated cells in cells carrying a CBF fusion than in cells without fusion independent of the fusion gene detected (Fig. S22). In one patient, we identified a tumor cell population harboring multiple tumor clones and a non-tumor cell population, harboring a clonal hematopoiesis-associated mutation that was stable during treatment (Fig. 1A, B).

**Fig. 1:**
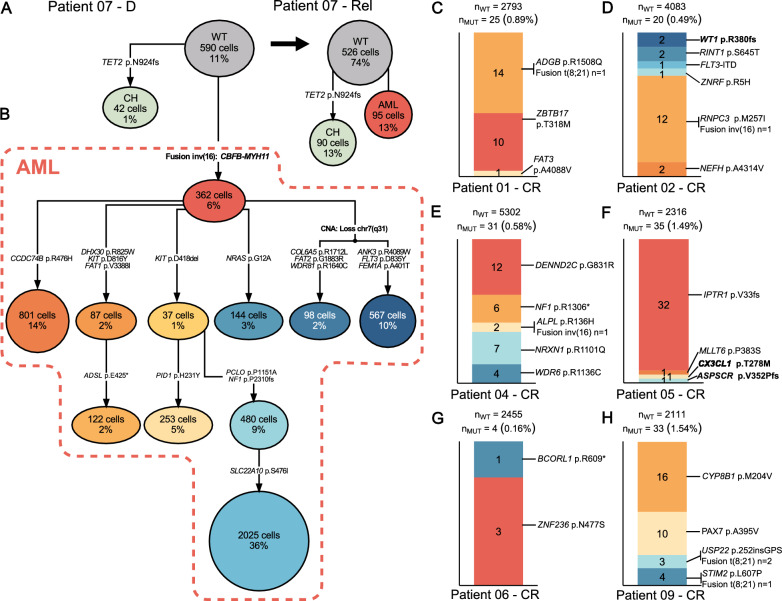
Persisting clones throughout treatment. Inferred phylogenetic tree of patient 07 with a (**A**) persisting clonal hematopoiesis (CH) clone at diagnosis and relapse with 42 cells (1%) and 90 cells (13%), respectively, and (**B**) the AML clone of the diagnosis sample. **C-H** Bar plots showing mutated cells detected in complete remission. Colors represent tumor clones from inferred phylogenetic trees of diagnosis sample (patient 04,06,09: Fig. S12) or diagnosis and relapse sample combined (patient 01,02,05: Fig. S13). Clones are labelled by mutation identified in the CR cells and highlighted in bold if relapse-specific. Detection of fusion genes is indicated next to the respective clones to which the cells were assigned, based on co-mutations

We used CR samples from 6 patients for tumor cell detection during molecular remission on single-cell level (confirmed by measurable residual disease (MRD) assessment via qPCR [12]). Remaining tumor cells that harbored ≥1 variant/fusion were identified in all CR samples (4–35 cells, 0.16%-1.54%, Fig. 1C–H). In 93 cells 1 variant/fusion was identified at CR, 55 cells carried >1 alteration (Figs. S23-S25). Applying the infinite-sites assumption [10], we assigned each cell to tumor clones from inferred phylogenetic trees from diagnosis or diagnosis and relapse. Among the 148 cells with detectable variant/fusion, 4 carried relapse-specific variants and only 6 cells carried the CBF fusion in CR (Fig. 1C–H). Of those patients with relapse samples available (in scDNA-seq or WES), the majority of CR variants (101/119) were detected at diagnosis and relapse indicating their presumed association with the CBF AML. Thus, the parallel assessment of multiple patient-specific genetic aberrations markedly enhanced the sensitivity of MRD detection relative to the exclusive targeting of CBF fusions in scDNA-seq.

Next, we modelled clonal evolution on single-cell level for three patients with available material for scDNA-seq from all timepoints and sufficient quality for phylogenetic analysis. Patient 01 lost the late diagnosis-specific *FLT3* D835 clones at relapse, which were also not present at CR (Fig. 2A–C). At relapse, patient 02 lost a diagnosis-specific branch while acquiring a *WT1* mutation (Fig. 2D–F). Patient 05 acquired 8 new variants/subclones at relapse (Fig. 2G–I). All three patients shared the founding and early acquired events between diagnosis and relapse, indicating similar clonal evolution patterns and incomplete eradication of disease initiating events.

**Fig. 2:**
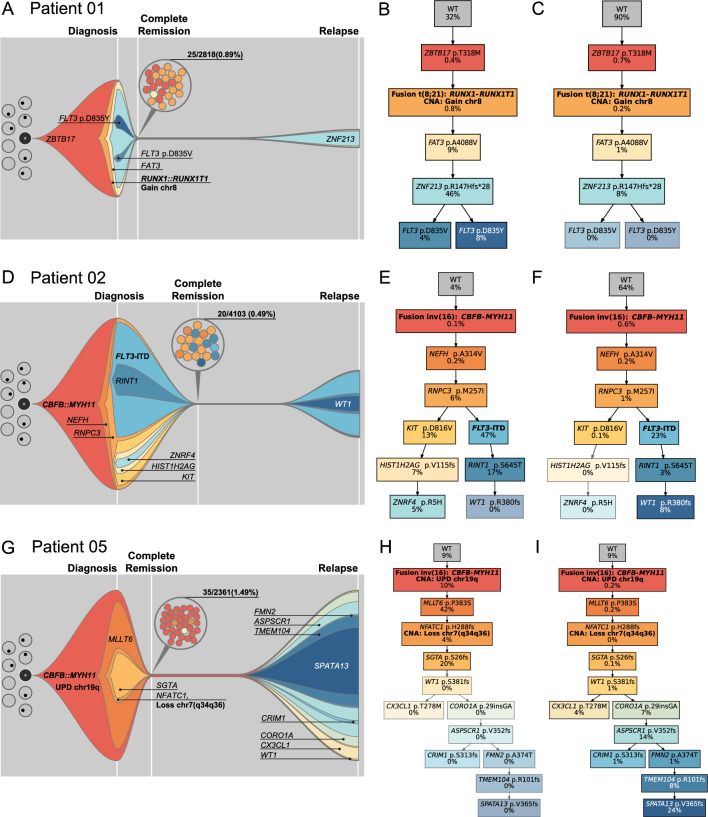
Clonal evolution of longitudinal CBF AML samples. Fish plots of **A** patient 01, **D** patient 02, **G** patient 05 including diagnosis (D), complete remission (CR) and relapse (Rel). Clone sizes are normalized to percentage of blasts in the sample. The grey background represents the wild-type cell fraction. Colored circles at CR represent the cell counts and assigned tumor clones as inferred by the infinite-sites model. **B, C** Simplified phylogenetic tree of patient 01 at diagnosis and relapse, respectively. **E, F** Simplified phylogenetic tree of patient 02 at diagnosis and relapse, respectively. **H, I** Simplified phylogenetic tree of patient 05 at diagnosis and relapse, respectively. The greyed-out clones are not present in the respective timepoint

Although, the described approach is labor intensive, the sensitivity of subclonal events is an advantage of the presented study. We included a high number of patient-specific somatic events resulting in detailed phylogenetic trees, resolving mutation order more precisely as compared to large-scale analyses restricted to few events per patient [13]. We integrated SCNAs not covered by SNVs into phylogenetic tree analysis and validated the results with karyotype data. With this approach we detected subclonal SCNAs that have been missed by conventional bulk sequencing methods, unravelling the complexity of the disease in detail. To adapt this method for clinical purposes, we suggest screening for therapeutic targets and AML drivers to determine the order of mutation acquisition.

In conclusion and with the limitation of a small patient cohort, our study highlights the necessity of identifying early events during tumorigenesis in CBF AML. Expanding the detection spectrum through the parallel analysis of multiple patient-specific co-occurring genomic aberrations (CBF fusions and mutations) enabled the identification of residual tumor cells in all patients during complete remission, underscoring the method’s technical utility and sensitivity for early detection of disease progression.

## Supplementary Information

Supplementary material 1. Document S1. Material and Methods, Extended Results, Tables S1–S5, S9 and Figures S1–S25.

Supplementary material 2. Table S6. Variant List. Variants identified via bulk sequencing (WES and targeted sequencing) and single-cell DNA sequencing.

Supplementary material 3. Table S7. Custom Targeted Single-Cell DNA Sequencing Panels. Custom targeted panels for MissionBio Tapestri single-cell DNA sequencing.

Supplementary material 4. Table S8. Single cell read counts. Reference and alternative read counts for known variants in single-cell DNA sequencing samples used as an input for inferring tumor phylogenies.

## Data Availability

Raw sequencing data generated during the current study have been deposited in the European Nucleotide Archive (ENA) at EMBL-EBI under accession number PRJEB75961 ([https://www.ebi.ac.uk/ena/browser/view/PRJEB75961]). The modified version of COMPASS is available under following GitHub repositories: [https://github.com/RaphaelHablesreiter/] (https://github.com/RaphaelHablesreiter/scPhyloSCNA).

## Abbreviations

ADO: Allele dropout
AML: Acute myeloid leukemia
CBF: Core-binding factor
CBF AML: Core-binding factor acute myeloid leukemia
CH: Clonal hematopoiesis
Chr: Chromosome
CR: Complete remission
ITH: Intra-tumor heterogeneity
qPCR: Quantitative polymerase chain reaction
scDNA-seq: Single-cell DNA sequencing
SCNA: Somatic copy-number alteration
SNV: Single nucleotide variant
WES: Whole exome sequencing
