# Supplementary material for: Resolving intra-tumor heterogeneity and clonal evolution of core-binding factor acute myeloid leukemia patients with single-cell resolution
Source: Exp Hematol Oncol. 2025 Oct 28;14:127. doi: 10.1186/s40164-025-00718-4 (PMC12570578; doi:10.1186/s40164-025-00718-4)
Supplement: Supplementary file 1 — Supplementary material 1. Document S1. Material and Methods, Extended Results, Tables S1–S5, S9 and Figures S1–S25. [file 40164_2025_718_MOESM1_ESM.docx]

**Supplementary Information**

**Resolving intra-tumor heterogeneity and clonal evolution of core-binding factor acute myeloid leukemia patients with single-cell resolution**

Raphael Hablesreiter^1^, Paulina M Strzelecka^1^, Klara Kopp^1^, Natalia Estrada^1^, Anna Dolnik^1^, Marlon Tilgner^1^, Coral Fustero-Torre^1^, Felicitas Thol^2^, Florian H Heidel^2,3^, Michael Heuser^2,4^, Laleh Haghverdi^5^, Lars Bullinger^1,6^, Friederike Christen^1*^ and Frederik Damm^1,6*^

1. Charité – Universitätsmedizin Berlin, corporate member of Freie Universität Berlin and Humboldt-Universität zu Berlin, Department of Hematology, Oncology and Cancer Immunology, Berlin, Germany
2. Department of Hematology, Hemostasis, Oncology and Stem Cell Transplantation, Hannover Medical School (MHH), Hannover, Germany.
3. Leibniz Institute on Aging, Fritz-Lipmann-Institute, Jena, Germany
4. Department of Internal Medicine IV, University Hospital Halle (Saale), Martin-Luther-University Halle-Wittenberg, Halle, Germany
5. Max-Delbrück-Center for Molecular Medicine in the Helmholtz Association (MDC), Berlin Institute for Medical Systems Biology (BIMSB), Berlin, Germany.
6. German Cancer Consortium (DKTK), partner site Berlin and German Cancer Research Center (DKFZ), Heidelberg, Germany

*FC and FD contributed equally as senior authors.

# Material and Methods

## Patients and Material

Two female and 7 male patients with CBF AML and available samples at diagnosis (D), complete remission (CR) and relapse (Rel) were investigated in this study. The age of those patients at diagnosis ranged from 30 to 67 years. Patients in this cohort were categorized according to the French-American-British (FAB) classification as patients with acute myelomonocytic leukemia (M4) (6/9), acute myeloblastic leukemia with maturation (M2) (2/9) and acute myelomonocytic/monocytic leukemia (M4/M5) (1/9). All patients reached CR and relapsed within 27 months. Overall survival (OS) ranged from 14 to 104 months. Five patients died (patients 01, 02, 03, 07 and 09) and 4 patients (patients 04, 05, 06 and 08) were censored at last follow-up (Table S1).

All patients received therapy as listed in Table S2, with 7 out of 9 patients receiving allogeneic stem cell transplantation as salvage treatment. Patients 05, 06, and 08 were enrolled in clinical trials. DNA was isolated from peripheral blood or bone marrow (Table S3) with commercially available kits. For patients without any DNA or cell material available from complete remission, flow-sorted T cells from diagnosis were used as non-tumor control for WES. Sample availability and sequencing status are reported in Table S3.

## Whole Exome Sequencing

WES libraries were generated using SureSelect All Exon v7 XT HS kit (Agilent, Santa Clara, CA, USA) according to the manufacturer’s instructions. Libraries were sequenced on an Illumina NovaSeq 6000 platform in 2x150 bp paired-end mode.

Variant calling was performed using the variant calling part of our in-house Snakemake-pipeline^1^ as previously described^2-6^. FASTQs were processed using Trimmomatic (v0.36)^7^ in paired-end mode with default parameters and “LEADING:3 TRAILING:3 SLIDINGWINDOW:4:15 MINLEN:36”. Subsequently, reads were aligned to hg19 human reference genome^8^ using bwa mem^9^ and samtools sort (v1.11)^10^. Polymerase chain reaction (PCR) duplicates were removed using Picard’s (v2.20.0)^11^ MarkDuplicates. These aligned and filtered reads were used for variant calling, *FLT3*-ITD detection and SCNA analysis. Variant calling was performed using the variant calling part of our in-house Snakemake-pipeline^1^ consisting of a preprocessing and variant calling part, as previously described^2-6^. VarDictJava (v 1.8.2)^12^ was executed on aligned reads in single-sample mode with a minimum VAF of 0.1%, the reference genome used in preprocessing, the bed file provided by Agilent (SureSelect All Exon v7 XT HS) and default parameters. Raw variant calls were processed using bcftools’ (v1.11)^10^ view, index and norm, and, subsequently, converted using R to the appropriate format for annotating them with ANNOVAR [version 2020-06-07 23:56:37 -0400 (Sun, 7 Jun 2020)]^13^ and following databases: refGene^14^, clinvar_2021050^15^, dbnsfp42c^16,17^, gnomad_exome^18^, avsnp150 (dbSNP140)^19^, cosmic70^20^, revel^21^, nci60, icgc28^22^, snp142^19^ and popfreq_all_20150413 (containing frequencies from 1000G, ESP6500, ExAC and CG46). Variants were further annotated with manually curated lists from our group for known AML driver or AML candidate genes and if the variant has been published as known CHIP (clonal hematopoiesis of indeterminate potential) variant or CHIP hotspot. Additionally, Fisher’s Exact Test as used in the Genome Analysis Toolkit (GATK)^23^ was calculated for each variant.

Variants were removed according to quality metrices for all samples of a patient using the following filtering criteria:

- VAF >1% at complete remission or in extracted T-cells from diagnosis
- VAF <4% (VAF <6% for patient 03) at diagnosis and relapse
- read depth <50 at diagnosis and relapse
- variant read count <6 at diagnosis and relapse
- FisherScore ≥20 at diagnosis and relapse
- StrandBalance1 or StrandBalance2 is 0|1|NA at diagnosis and at relapse

Additionally, synonymous and non-frameshift variants, variants with a minor allele frequency (MAF) >0.01% in the gnomad_exome^18^, avsnp150 (dbSNP140)^19^ or popfreq_all_20150413 (PopFreqMax)^13^ were filtered if not flagged as important. A variant was flagged as “important” if the gene is (i) a AML candidate or (ii) a AML driver gene, the variant (iii) is a known CHIP mutation or (iv) is associated with hematopoietic diseases according to the COSMIC database ^20^. The remaining candidates were manually filtered by visual inspection using Integrative Genomics Viewer (IGV) (v 2.11.6)^24^.

ITDetect (v.1.4)^25^ was performed on all BAM files to detect *FLT3* internal tandem duplication (ITD) with the chromosomal location of *FLT3* (chr13:28608020-28608360) and default parameters.

Somatic copy number alterations (SCNAs) were detected using refphase (v0.1.1)^26,27^ with ASCAT (v3.1.0)^28^ according to the ”Complete Example Workflow” in the refphase repository and, subsequently, manually filtered by inspecting B-allele frequency (BAF) from heterozygous single nucleotide polymorphisms (SNPs) and the log read-depth ratio (LogR) for each tumor sample. Here, positions from the dbSNP database (build: 151, reference: GRCh37.p13)^19^ were used to pileup reads using bcftools’ (v1.11)^10^ mpileup and bcftools’ query for each sample. These tables were processed using R and following libraries: ASCAT (v3.1.0)^28^, refphase (v0.1.1)^26,27^, dplyr (v1.1.1)^29^, tidyr (v1.3.0)^30^, glue (v1.6.2)^31^, gtools (v3.9.4)^32^ and readr (v2.1.4)^33^. Only positions on autosomes and with a minimum read count of 50 reads for reference and alternative reads combined were used. Identified candidates for SCNAs were manually filtered.

## Targeted Sequencing

Targeted Sequencing libraries were generated as described previously^2-5^, using a 45 gene-panel (Table S4). In brief, library construction was performed using a hybrid-capture based library preparation kit (TWIST Bioscience, South San Francisco, CA, USA) and unique molecular identifiers (UMIs, xGen UDI-UMI adapters by Integrated DNA Technologies, Coralville, IA, USA). Libraries were sequenced on a NovaSeq 6000 platform (Illumina, San Diego, CA, USA) in paired-end mode (2x 150bp).

Aligned reads were generated using the preprocessing part of in-house Snakemake-pipeline^1^. Initially, Picard’s (v2.20.0)^11^ ExtractIlluminaBarcodes and IlluminaBasecallsToSam to extract unmapped BAM (uBAM) files storing the UMI sequence as RX SAM tag from Illumina basecalls. uBAMs were aligned to hg19 human reference genome^8^ using Picard’s SamToFastq, bwa mem (v0.7.17)^9^ and Picard’s MergeBamAlignment subsequently. fgbio’s (v 0.6.1)^34^ GroupReadsByUmi and CallMolecularConsensusReads were performed to group aligned reads based on UMIs and to further create consensus reads with a minimum of 3 supporting UMIs. These consensus reads were aligned again to the reference genome and further quality filtered using fgbio’s FilterConsensusReads with a minimum of 3 supporting UMIs, consensus bases with a quality >5 and default parameters.

Variant calling was performed as described for WES data, but with a minimum VAF of 0.01%. Raw variants were filtered for following quality criteria in each patient, except for patients 06 and 08 where criteria were applied to diagnosis and, respectively, relapse only:

- VAF >1% at complete remission
- VAF <2% at diagnosis and relapse
- read depth <50 at diagnosis and relapse
- variant read count <4 at diagnosis and relapse
- FisherScore ≥20 at diagnosis and relapse
- StrandBalance1 or StrandBalance2 is 0|1|NA at diagnosis and at relapse

Additionally, synonymous and non-frameshift variants, variants with a minor allele frequency (MAF) >0.01% in the gnomad_exome^18^, avsnp150 (dbSNP140)^19^ or popfreq_all_20150413 (PopFreqMax)^13^ were filtered if not flagged as important. A variant was flagged as “important” if the gene is (i) a AML candidate or (ii) a AML driver gene, the variant (iii) is a known CHIP mutation or (iv) is associated with hematopoietic diseases according to the COSMIC database^20^. The remaining candidates were manually filtered by visual inspection using Integrative Genomics Viewer (IGV) (v 2.11.6)^24^.

## Nanopore sequencing

Nanopore libraries were prepared using the ligation sequencing kit SQK-LSK110 (Oxford Nanopore Technologies, Oxford, UK) and sequenced on a GridION Mk1 (Oxford Nanopore Technologies, Oxford, UK) using MinION flow cells FLO-MIN106D (Pore: R 9.4.1) (Oxford Nanopore Technologies, Oxford, UK). Nanopore reads were aligned to the humanG1Kv37 reference genome^35^ using Vulcan (v1.0.3)^36^ with default parameters. NanoFG (v1.0)^37^ was performed on the mapped reads to get a FASTA sequence flanking ±400 bp of the breakpoint sequence and default parameters. For patient 03 the “do not filter”-option was additionally used to get a result.

## Nuclei Extraction for Single-Nuclei DNA Sequencing

For patient 05, single cell sequencing of viable PBMC yielded low quality output, therefore we went for nuclei extraction from frozen cells. The extraction was performed as recommended by MissionBio. In brief, frozen cells were transferred into a pre-chilled petri-dish on dry ice and incubated with tissue lysis solution. The cells were minced with a pre-chilled scalpel while bringing to room temperature. The tissue was lysed for 15 min at RT before stopping the reaction with Trypsin inhibitor solution. Nuclei were filtered through a cell strainer and quantified via DAPI staining in an automated cell counter (Countess 3 FL, Thermo Fisher Scientific) using a DAPI LED cube (Thermo Fisher Scientific). The samples were diluted to 3000 nuclei/µl in Mission Bio cell buffer and subjected to the Tapestri protocol.

## Single-cell DNA sequencing

Single cell DNA sequencing was performed for peripheral blood or bone marrow mononuclear cells on the MissionBio Tapestri platform V2 using the Tapestri Single-Cell DNA sequencing Kit V2 (MissionBio, South San Francisco, CA, USA). Three different custom targeted panels were designed by MissionBio based on whole exome sequencing, targeted sequencing and nanopore sequencing data (Table S6 and S7).

For patients 06, 08 and 09 no relapse sample was available for scDNA-seq and for patients 07 and 08, we did not have viable cells from CR for scDNA-seq (Table S3).

For one sample, library prep was performed with extracted nuclei instead of peripheral blood mononuclear cells due to limited number of viable cells after thawing (see above). Libraries were sequenced on Illumina’s NovaSeq 6000 platform (Illumina, San Diego, CA; USA) using an SP or S1 flow cell in 2x 150 bp paired-end mode with 15% PhiX. Sequencing reads were processed using the Tapestri pipeline (Mission Bio, v2.0.2) with the respective panel and custom reference genome to obtain loom files that were used for downstream analysis. The custom reference genome contains autosomes, sex chromosomes and, additionally, the sequences ±400 bp around fusion gene breakpoints of patients included in the panel.

Information on variants, CBF gene fusion and cell barcodes was retrieved using an adapted preprocessing script from COMPASS (v.1.1)^38^ (Table S8). Genotypes were annotated as 0 for wild-type, 1 for heterozygous, 2 for homozygous and 3 for missing. Genotypes for each variant in each cell were additionally filtered and set to missing when: (i) the genotyping quality (GQ) is <15, (ii) the depth is <6 reads or (iii) the genotype is heterozygous or homozygous with an allelic frequency (AF) <20%. A read that maps to the patient specific gene fusion, which have been added as additional sequences to the reference genome, is counted as one reference and one alternative allele count (i.e. annotated as heterozygous). In case of patients with two detected breakpoints in bulk (Table S5) and, therefore, two sequences in the reference genome, read counts on both amplicons were summarized. *FLT3*-ITD was detected within a cell for patient 02 when the alternative allele consisted of at least 2 nucleotides in amplicon AMPL135278, which is the amplicon specifically designed for *FLT3*-ITD of patient 02. To compare VAFs of detected variants between bulk and single-cell sequencing (Figure S11), we inferred single-cell VAFs from genotypes (Table S6) using the following formula:

${VAF}_{single-cell}=\frac{n_{HOM,cells}+n_{HET, cells}\times0.5}{n_{total cells}}$.

The number of heterozygous cells is weighted by a factor of 0.5, due to the assumption that the VAF of a heterozygous cell is ideally 50%. For evaluating the specificity of the fusion gene amplicons, cell barcodes for each read mapping to a fusion gene amplicon were extracted and processed using R (Figure S15). Cell barcodes were filtered for barcodes identified in the variant detection step.

## Reconstruction of phylogenetic trees

For phylogenetic tree reconstruction, we excluded variants with low coverage (<20% cells with a minimum depth of 15 reads), when less than 5 mutated cells were detected at the time of diagnosis or when the number of mutated cells did not differ between diagnosis and complete remission samples (used for phylogenetic tree: no, Table S6). We inferred phylogenetic trees using a 2-step approach, to detect SCNAs independent of LOH and SNV sites:

(i) Tumor phylogenies from selected variants and gene fusions were inferred using COMPASS with the corresponding sex, with 10 Markov-Chain Monte-Carlo chains in parallel and 20,000 iterations in each, without SCNAs and default parameters. The phylogenies were inferred from the number of reads supporting the reference and alternative allele for each variant in a cell and not inferred from genotypes.

(ii) For the analysis of SCNAs of each tumor clone in an inferred phylogenetic tree, we used R and Python 3 (v3.7.9)^39^ with following packages: reticulate (v1.28)^40^, ggplot2 (v3.4.4)^41^, data.table (v1.14.8)^42^, stringr (v1.5.0)^43^, ggpubr (v0.6.0)^44^, dplyr (v1.1.1)^29^, jsonlite (v1.8.5)^45^, readxl (v1.4.2)^46^, ggh4x (v0.2.6)^47^ and mosaic (v2.2) (MissionBio, South San Francisco, CA, USA).

Each custom panel contains specifically designed amplicons for copy-number analysis that are grouped by chromosomes and annotated as region of interest (ROI), covering a segment of a known SCNA and copy-number neutral regions. If a ROI covers a chromosome only partially, amplicons on that chromosome are split into regions left and right of the ROI and the ROI itself. For each amplicon the fraction of cells that have a depth of ≥30 reads is calculated and if this fraction is ≤75% the amplicon is removed from further analysis. For amplicons in ROIs this threshold is relaxed to ≤50%, because in ROIs more amplicons allow for better SCNA estimation. Copy-number neutral regions were excluded from further analysis if they consisted of less than 4 amplicons. This set of amplicons grouped into copy-number neutral regions and ROI is used for detecting SCNAs.

For the identification of SCNAs in tumor clones, cell assignments from COMPASS were used to remove doublets and to assign cell barcodes to tumor clones and wild-type cell fraction of the phylogenetic tree. Based on this, ploidies *p_ij_* for each cell *j* at amplicon *i* were calculated with the wild-type fraction as diploid reference. The variance Var(p_ij_) and Z-scores Z_ij_ for ploidies *p_ij_* were calculated. Subsequently, ploidies of copy-neutral regions and ROIs were determined using cells with Z_ij_<2 and a weighted mean so that higher variance amplicons *i* contribute less to the region ploidies.

For tumor clones with SCNAs the ploidies can be elevated across all regions. Therefore, ploidies of regions were centered by subtracting the mean of the ploidies of copy-neutral regions (R1, R2, …) ‑2 (=ploidy of copy-neutral region) to counteract the elevated ploidies.

Uniparental disomys (UPDs) were detected by using genotype information of single nucleotide polymorphisms (SNPs) and the resulting fractions of wild-type, heterozygous and homozygous cells for each clone.

To assure stable phylogenetic trees, we inferred tumor phylogenies with 10 restarts, different parameter settings of COMPASS and removed or added variants/SNPs manually. We used different parameters for the number of chains (*i.e.*, 5,10 and 20) and the number of iterations (*i.e.*, 5,000, 10,000, 20,000 and 40,000) of the Markov-Chain Monte-Carlo chains. Additionally, we used the copy-number alteration detection method of COMPASS to ensure stable inferred phylogenetic trees of all samples. In case of clonal evolution analysis, we inferred trees for each timepoint individually prior to the combined analysis of diagnosis and remission samples to assure tree stability.

We have generated heatmaps for samples at diagnosis (Figures S16 and S17) and for combined samples from diagnosis and relapse to illustrate the clonal structure of the data (Figures S18-S21). We used the genotype information from Table S8 as input and partitioned the columns/cells based on the inferred clones from COMPASS as shown in Figures S13 and S14. Additionally, we excluded cells flagged as duplicates by COMPASS. In case of combined samples, we generated heatmaps for the combined cells and heatmaps separated by cell origin. Each variant in each cell is classified as wild-type (WT), heterozygous (HET), homozygous (HOM) or missing (MISS). Additionally, a missing (MISS) genotype does not exclusively indicate that a variant is absent in a cell due to lack of coverage, it can also mean that the variant cannot be confidently classified as WT, HET or HOM. For the combined samples of patient 01, we subset the heatmap to display only the clones preceding and including the first clone harboring the *RUNX1::RUNX1T1* fusion (Figure S21A). The same approach was applied to patient 09 at diagnosis (Figure S21B).

## Detecting clones in remission

For each complete remission sample, cells with at least one variant or gene fusion were selected for downstream analysis using R. The infinite-sites model allows every variant in a phylogenetic tree to change only one time from wild-type to mutated and is never lost^48^. Based on the infinite-sites model, the selected cells have been assigned to the tumor clone that had matching somatic variants and was the furthest away from the wild-type fraction. Results were visualized using R with packages ggplot2 (v3.4.4)^41^ and latex2exp (v0.9.6)^49^.

# Extended Results

## Somatic Mutation Detection by single-cell DNA sequencing

We identified 405 variants via bulk sequencing. In the diagnosis samples (n=9) 232 variants were detected and 173 variants in the relapse samples (n=8, no relapse sample was available for patient 06), resulting in 25.8 and 21.6 mean variants per patient, respectively (Figures S1, S2). The lower number of variants as well as the lower variant allele frequencies (VAF) in the relapse samples (Figures S3, S4A, Wilcoxon signed-rank test, p<0.001) is in line with the lower blast counts in relapse samples (Table S3, Figure S4B, p=0.021 Wilcoxon signed-rank test).

The most recurrently mutated gene at diagnosis was *FLT3* (4 out of 9 patients, 44%, Figure S2). In three patients we observed mutations *AZGP1* and *KIT* at the time of diagnosis. At relapse, we did not find any *FLT3* mutations using WES or targeted sequencing. The most recurrently mutated gene at relapse was *WT1*, which we exclusively found in the relapse samples from 4 out of 8 patients (50%). Patient 03 had the highest number of mutations in both samples (Figure S1, Figure S2 upper panel), while in patient 05, we detected the highest proportion of frameshift substitutions, especially in the relapse sample (Figure S2 upper panel).

We identified 7 SCNAs in diagnosis and relapse samples of 5 patients via WES. Patients 02 and 06 did not have any copy number alterations. Patient 03 and the relapse sample of patient 01 were excluded from SCNA analysis due to poor quality. In patient 01, we confirmed a gain of chr 8 in the diagnosis sample (Figure S5A) that was also detected via conventional karyotyping (Table S1). Patient 05 had a subclonal deletion in chr 7 and a UPD of chr 19 that expanded from diagnosis to relapse (Figure S5B). Patient 07 acquired an additional SCNA at relapse (amplification in chr 9, Figure S5C), that was not detected at diagnosis. Patient 08 lost the gain of chr 22 that was identified at diagnosis in the relapse sample (Figure S5D). In patient 09 the UPD in chr17 was more pronounced at diagnosis (Figure S5E). These SCNA were included in the amplicon panel for scDNA-seq to unravel the clonal acquisition of these events.

We sequenced a median of 4103 cells/sample by scDNA-seq (range: 711-7560) with a mean coverage of 106 reads/amplicon/cell (range: 35-384, Figures S6-S9, Tables S8 and S9). In total, 514 out of 571 amplicons (90%) passed the coverage threshold (>0.2*mean reads/amplicon/cell, Figures S7 and S8).

In the 14 diagnosis and relapse samples with available material for scDNA-seq (Table S3), we identified 362 variants via bulk sequencing. For 9 of 362 variants (at 6 distinct positions) no amplicon could be designed for scDNA-seq (*CEBPA* p.P175R, *CEBPA* p.A135T, *IDH2* p.R18P, *MAP3K21* p.E50Q, *PTPN20* p.G28E, and *RGPD8* p.D1388Y, Table S6). Excluding these positions, we were able to detect 241 out of 353 variants identified via bulk sequencing with the custom scDNA-seq panels, leading to 28-96% of variants successfully identified per patient (median: 87%, Figure S10). The VAFs inferred from scDNA-seq genotyping showed a high correlation with the VAFs from bulk sequencing (R=0.82, p<0.001, Figure S11A), indicating that the sequenced single cells are representative of the bulk sample. Inspecting variants with low VAFs (Figure S11B), bulk VAF seems to be estimated higher than single-cell VAF, likely due to the reason that scVAF was estimated based on the cell genotype rather than the alternative and reference reads per variant in all cells. In the samples of patient 03, we could not confirm 79 of 109 variants previously identified via bulk sequencing due to poor sample quality.

We identified 7 additional variants by scDNA-seq in the regions covered by the custom amplicon panels that have not been detected in bulk sequencing. However, variants were detected in a different time point of the same patient. Further, the variants detected only in scDNA-seq but not in bulk sequencing were variants with VAFs ≤5% and *ZNF213* being detected with a depth of only ~100x, reducing the probability of detecting a variant with a 5% VAF. Unlike variant calls from bulk sequencing, where low variant allele frequencies (VAFs) often fall within the range of sequencing artefacts, mutations identified in single cells exhibit a VAF of at least 20% in each cell (Figure S12). This underscores the advantage of single-cell methodologies in reliably detecting low-frequency variants that may be missed by bulk sequencing approaches.

## Patient-specific fusion gene detection and tumor phylogeny at diagnosis

Fusion gene breakpoints were identified for every patient by nanopore long-read sequencing (Table S5). Sequences ±200bp around the breakpoint were included in panel-specific reference genomes for single-cell analysis. Fusion genes were successfully identified in single cells for 8 out of 9 patients using patient-specific amplicons. For patient 03, no reads were aligned to the fusion gene sequence (Figure S15). Because of this and the low variant overlap between bulk und scDNA-seq, we excluded patient 03 from phylogenetic and later clonal evolution analysis.

To estimate timing of CBF fusion acquisition during leukemogenesis, we classified single cells based on their fusion gene status (yes, if ≥3 reads mapped to the patient-specific gene fusion, Figure S15). We calculated the percentage of cells that harbored at least one hetero- or homozygous mutation for each variant. At diagnosis a median of 12% of cells without fusion reads were mutated, whereas for cells with fusion reads a median of 81% were mutated. At relapse a median of 2.2% of cells without fusion reads were mutated and 76% of cells positive for fusion reads (p<0.001 for both time points, Wilcoxon signed-rank test, Figure S22). This indicates that CBF fusion genes are acquired earlier than other variants. We estimate that the percentage of mutated cells without fusion reads accounts for the dropout rate of CBF amplicons, which are more complex and therefore harder to detect even in scDNA-seq. Of note, Sollier *et al.*^38^ have shown that the allelic dropout (ADO) rates of each allele are around 5%, but vary a lot when using the 50-amplicon panel from Morita *et al.*^50^. The median ADO rate in our samples ranges from 12.9% to 21.8% for heterozygous SNPs located in regions of amplicons that were designed for copy-number analysis (Figure S9). We did not incorporate specific amplicons for heterozygous SNPs in every panel (Table S7) and, therefore, we cannot compare the ADO of the same amplicons across all samples. The low percentage of wild-type cells in the diagnosis sample might also lead to an overestimation of mutated cells without fusion reads.

For the diagnosis samples of patients 02 and 06, we inferred tumor phylogenies based on somatic variants and gene fusions, resulting in trees consisting of 5 and 3 tumor clones, respectively. These patients did not have any SCNAs. In patient 02, two distinct branches evolved from the gene fusion-carrying founding clone in parallel: one with *KIT* p.D816V driver mutation and another with *FLT3*-ITD (Figure S13B). Patient 06 had the least complex phylogenetic tree consisting of only three tumor clones: the founding clone with the *CBFB*::*MYH11* gene fusion (123 cells) and two distinct subclones: one with a dominant tumor clone harboring *NRAS* p.Q61H (83% of all cells, n=2179) and a second one with a minor subclone characterized by a *BCORL1* mutation (207 cells, Figure S13E). Of note, patient 06 had the longest OS of all investigated patients (censored at 104 months, Table S1).

## Inferred tumor phylogenies at diagnosis using SNVs and SCNAs

For 6 patients, we additionally detected SCNA that were incorporated into the phylogenetic tree together with somatic variants and CBF gene fusions. We were able to detect all previously identified SCNAs in single cells (including amplifications, deletions and UPDs) and included them in the tumor phylogenies. Additionally, we detected amplifications that were not detectable by WES. Here, we want to highlight that COMPASS uses a probabilistic generative process for inferring the phylogenetic trees from read count data and not from the genotype of a variant within a cell. Additionally, a MISSING genotype not exclusively indicates that a variant is absent in a cell due to lack of coverage, it can also mean that the variant cannot be confidently classified as wild-type (WT), heterozygous (HET) or homozygous (HOM). This also means that by presenting only the genotypes we cannot account for doublets and, therefore, we cannot remove them.

In the diagnosis sample, patient 05 showed a linear phylogenetic tree, with the *CBFB::MYH11* fusion and a UPD of chr 19q as part of the founding clone (Figure S13D). Here, the majority of the cells acquired a single somatic mutation (*ELTD1*, n=3070) in addition to the founding clone events. Partial loss of the short arm of chr 7 was found in 29% of the cells confirming karyotyping (13/46 metaphases, Table S1) and WES results (Figure S5B).

The patients with *RUNX1::RUNX1T1* fusion (patients 01 and 09) revealed phylogenetic trees with 5 and 7 clones, respectively. The *RUNX1::RUNX1T1* fusion was acquired subclonal after linear acquisition of somatic mutations, indicating a higher number of early non-driver mutations in patients with t(8;21). Both patients are characterized by late branching into two subclones. In patient 01, the *RUNX1::RUNX1T1* fusion was acquired together with a copy number gain of chr 8 (Figure S13A). From the major subclone, carrying an additional *ZNF213* frameshift mutation (45% of cells, n=1396), two distinct *FLT3* D835 subclones emerged in parallel (D835Y with 241 cells and D835V with 126 cells). In patient 09, three small subclones were acquired in a linear fashion (Figure S13H). The acquisition of the *RUNX1::RUNX1T1* fusion was followed by a LOH in chr 22 and a late branching into a major subclone with 1425 cells and a minor subclone carrying a *KRAS* G13D driver mutation (64 cells).

The founding clone of patient 08 at diagnosis harbored the *CBFB::MYH11* gene fusion followed by two distinct branches (Figure S13G). The major clone carrying a *FLT3* p.A680V mutation (70% of all cells) additionally gained an amplification of chr 22. Interestingly, only variants of the founding clone were detected in bulk sequencing. The amplification of chr 22 was also unique to diagnosis.

The inferred phylogenetic tree from patient 04 at diagnosis consisted of 6 tumor clones with the founding clone harboring the *CBFB::MYH11* gene fusion (Figure S13C). The *NF1* clone with 24% of all cells (n=362) showed amplifications on chr 13, 14 and 22. This matched the number of metaphases (13/49, 27%) with one additional chromosome detected by conventional G-banding (Table S1). These amplifications were not detectable by WES.

In case of patient 07, we detected clonal hematopoiesis (CH) consisting of 42 cells with a *TET2* p.N924fs mutation that was not part of the AML clone (Figure S13F). This *TET2* mutation has been detected using error-corrected targeted sequencing and would have been missed using only WES due to its low VAF. This patient has the most complex phylogenetic tree of this cohort, consisting of 11 tumor clones. From the founding clone, harboring the *CBFB::MYH11* gene fusion two *KIT* variants (*KIT* p.D816Y and *KIT* p.D418del), *NRAS* p.G12A and *FLT3* p.D835Y evolve into distinct subclones, with some of the subclones acquiring additional somatic variants in AML driver genes. Due to the low number of cells in the relapse sample of patient 07, we excluded the sample from clonal evolution analysis. Nevertheless, we were still able to show that the CH clone persisted throughout the course of the disease (Figure 1A). This patient with the most complex clonal composition was also the patient with the shortest OS time (14 months, Table S1).

Our 2-step approach for identifying SCNAs in inferred tumor phylogenies utilizes amplicons in regions of SCNAs and copy-neutral regions, which allows us to call SCNAs that are not supported by SNVs. This approach further can detect SCNAs spanning larger section of a chromosome and whole chromosomes. The use of weighted means reduces error from noise of scDNA-seq data, such as amplification errors (Figure S7) and errors from ADO (Figure S9).

## Detecting Clones in Remission

We used available CR samples from 6 patients for the detection of remaining tumor cells during molecular remission as confirmed by molecular measurable residual disease (MRD) assessment using qPCR^51^. We identified remaining tumor cells that harbored at least one somatic variant or the CBF fusion in all available remission samples (excluding patient 03) ranging from 4 to 35 cells (0.16%-1.54%, Figures 1C-H). Among the 148 cells with any detectable variant/fusion, only 6 cells were positive for *CBFB::MYH11* or *RUNX1::RUNX1T1*. In 93 cells 1 variant/fusion was identified at CR, 55 cells carried >1 alteration (Figure S23).

By applying the infinite-sites assumption^52^ we assigned each cell to tumor clones from inferred phylogenetic trees from diagnosis (patients 04, 06 and 09, Figure S13) or diagnosis and relapse combined (patients 01, 02 and 05, Figure S14). In case of patient 01 (Figure 1C, S24A), we detected remaining tumor cells that have been assigned to tumor clones shared between diagnosis and relapse but were rather early developing clones (tier 1-3). Interestingly, for patient 02 (Figure 1D, S24B), we were able to assign remission cells to diagnosis (*ZNRF4* p.R5H) and relapse-specific tumor clones (*WT1* p.R380fs), indicating that both branches have been present in the patient during the whole course of the disease. For patient 04 (Figure 1E, S24C), remaining tumor cells were assigned to all tumor clones at diagnosis except to the *MALRD1* p.R944 clone, which was the clone that emerged last in tumor development of this patient. We detected the highest number of mutated cells (n=35) for patient 05 (Figure 1F, S24D) with the majority assigned to the founding clone (n=32). CR cells of patient 09 had the highest number of alterations (mean=3.4, Figure S17, S24B). In patients 02 and 05, two cells each harbored mutations associated with relapse-specific clones (Figure 1D and F, indicated in bold text). In summary, we could detect residual tumor cells in all patients with available CR samples. Of those patients with relapse samples available (in scDNA-seq or WES), the majority of variants at CR (101/121) were detected at diagnosis and relapse indicating their presumed association with the CBF AML. Thus, the parallel assessment of multiple patient-specific genetic aberrations markedly enhanced the sensitivity of MRD detection relative to the exclusive targeting of CBF fusions in scDNA-seq.

## Clonal evolution of CBF AML

We were able to infer clonal evolution on single-cell level for patients 01, 02 and 05 from merged samples at D, CR and Rel. Clonal evolution analysis of patient 01 revealed a similar clonal composition at diagnosis and relapse with the difference that both *FLT3* D835 clones were not present at Rel (Figure 2A-C, Figure S14A). As described in the previous section, these clones were also not detectable as residual tumor cells in remission (Figure 1C), indicating eradication of these clones by chemotherapy.

The combined phylogenetic tree of patient 02 consists of a diagnosis specific branch defined by *KIT* p.D816V, *HIST1H2AG* p.V115Rfs*23 and *ZNRF4* p.R5H subclones and a second branch with a dominant *FLT3*-ITD clone and a *RINT1* p.S304T clone (Figure 2D and E, Figure S14B). The *KIT* branch was lost, and the leukemia relapsed by acquiring two additional variants (*i.e.*, *WT1* p.R368Afs*5 and *ASAP1* p.N369S, Figure 2F). The somatic variants in the diagnosis-specific *KIT* branch were also not detectable in bulk sequencing data of the relapse sample. It has to be noted that the inferred phylogenetic trees from diagnosis alone (Figure S13B) and combined analysis of diagnosis and relapse samples are slightly different due to the increased number of cells in the combined analysis. The resolution of the combined analysis is higher and reveals more subclones.

In case of patient 05, the inferred clonal evolution showed that all tumor clones detected at diagnosis remained throughout therapy and progressed by gaining relapse specific variants (Figure 2G-I, Figure S14C). This was the only patient, with a similar tumor content at diagnosis and relapse, indicating progression of the disease at relapse.

In general, we observed stable clonal evolution in diagnosis and relapse, especially in the early occurring clones, for all three patients investigated here. Although some branches were lost after chemotherapy or acquired during relapse, the first events remained stable. This is in line with the observation that we observed a substantial number of remaining tumor cells in remission, indicating that relapse was driven by tumor cells that were not completely eradicated.

# Supplementary Tables

**Table S1**: Baseline patient characteristics. Survival times are given in months. Karyotype was determined via G-banding. *FLT3* internal tandem duplication (ITD) was detected via Genescan-based fragment analysis.

| Patient | Sex | Age | ECOG performance status | FAB classification | RFS time | OS time | Death | Karyotype | CBF | FLT3-ITD |
| --- | --- | --- | --- | --- | --- | --- | --- | --- | --- | --- |
| 01 | Male | 44 | 0 | M2 | 24 | 35 | yes | 47, XY, +8, t(8;21)(q22;q22), inv(9)(p11 q12) | t(8;21) | WT |
| 02 | Male | 48 | 0 | M4 | 4 | 53 | yes | 46, XY, inv(16) | inv(16) | MUT  (AR 0.13) |
| 03 | Male | 56 | 0 | M4 | 14 | 33 | yes | 46, XY, del11, inv(16), +8 | inv(16) | WT |
| 04 | Male | 30 | 0 | M4 | 9 | 22 | no | 46, XY, inv(16) (p13q22) 7/47, idem, +22 1/49, idem, +13, +14, +22 13/49. nuc ish 16q22  (CBFBx2)(5´CBFBsep3´CBFBx1) 99/100,11q23(MLLx2), 3q2(EVI1x2) 100 | inv(16) | WT |
| 05 | Male | 65 | 1 | M4/M5 | 14 | 79 | no | 46, XY, inv(16)(p13q22) 7/46, idem, del(7)(q31q33) 13/46. nuc ish 3q26(EVI1x2), cen7(CEP7x2), 7q31(D7S486x2), 11q23(MLLx2) 100, 16q22(CBFBx2) 96/100 | inv(16) | WT |
| 06 | Male | 36 | 0 | M4 | 1 | 104 | no | 46, XY, inv(16)(p13q22) | inv(16) | WT |
| 07 | Male | 67 | 0 | M4 | 9 | 14 | yes | 46, XY, del(16)(p12), inv(16)(p13q22) 3/46, idem, +(3;12)(q12;p13), add(19)(q13) 5/46,idem, der(X)t(X;17)(p11;q11), del(5)(q23q34), +mar 4/46, idem, add(7)(q31) | inv(16) | WT |
| 08 | Female | 52 | 0 | M4 | 14 | 80 | no | 47, XX, inv(16), +22 | inv(16) | WT |
| 09 | Female | 56 | 1 | M2 | 27 | 36 | no | 46, XX, t(8;21) | t(8;21) | WT |

ECOG: Eastern Cooperative Oncology Group, FAB: French-American-British, RFS: relapse-free survival, OS: overall survival, CBF: core binding factor, AR: allelic ratio; WT: wild-type, MUT: mutated

**Table S2:** Treatment regimens for all patients.

| **Patient** | **FAB** | **Phase** | **Treatment** |
| --- | --- | --- | --- |
| 01 | M2 | Induction | Idarubicin, Cytarabine, Etoposide |
|  |  | Consolidation | High-dose Cytarabine |
|  |  | Salvage | Mitoxantrone, Topotecan, Cytarabine + alloSCT |
| 02 | M4 | Induction | Idarubicin, Cytarabine, Etoposide, all-trans retinoic acid |
|  |  | Consolidation | High-dose Cytarabine |
|  |  | Salvage | Mitoxantrone, Topotecan, Cytarabine + alloSCT |
| 03 | M4 | Induction | Idarubicin, Etoposide, intermediate-dose Cytarabine |
|  |  | Consolidation | High-dose Cytarabine |
|  |  | Salvage | Mitoxantrone, Topotecan, Cytarabine + alloSCT |
| 04 | M4 | Induction | Daunorubicin, Cytarabine |
|  |  | Consolidation | High-dose Cytarabine |
|  |  | Salvage | High-dose Cytarabine, Mitoxantrone + alloSCT |
| 05 | M4/M5 | Induction | Idarubicin, Cytarabine, Etoposide |
|  |  | Consolidation | High-dose Cytarabine |
|  |  | Salvage | Fludarabine, Cytarabine, Idarubicin |
| 06 | M4 | Induction | Daunorubicin, Cytarabine |
|  |  | Consolidation | High-dose Cytarabine |
|  |  | Salvage | High-dose Cytarabine, Mitoxantrone + alloSCT |
| 07 | M4 | Induction | Daunorubicin, Cytarabine |
|  |  | Consolidation | High-dose Cytarabine |
|  |  | Salvage | - |
| 08 | M4 | Induction | Daunorubicin-Cytarabine |
|  |  | Consolidation | High-dose Cytarabine |
|  |  | Salvage | Cytarabine, Vosaroxin/Placebo + alloSCT |
| 09 | M2 | Induction | Idarubicin, Etoposide, intermediate-dose Cytarabine |
|  |  | Consolidation | High-dose Cytarabine, Daunorubicin |
|  |  | Salvage | - |

FAB: French-American-British classification, alloSCT: allogeneic stem cell transplantation

**Table S3:** Patient sample specifications and available material. The gene fusion was detected via qPCR. If DNA was available, whole exome and error-corrected targeted sequencing was performed. If cells were available, single-cell DNA sequencing was performed and the status of the scDNA-seq is reported as yes/no or fail. Failed samples were not included in the phylogenetic analysis.

| **Pat** | **Timepoint** | **Source** | **Blasts [%]** | **Gene fusion** | **Material availability** | | **Sequencing performed** | | |
| --- | --- | --- | --- | --- | --- | --- | --- | --- | --- |
|  |  |  |  |  | DNA | Cells | WES | Targeted Seq | scDNA seq |
| 01 | D | BM | >50 | positive | yes | yes | yes | yes | yes |
|  | CR | BM | <5 | negative | yes | yes | yes | yes | yes |
|  | Rel | BM | 30-40 | positive | yes | yes | yes | yes | yes |
| 02 | D | PB | 84 | positive | yes | yes | yes | yes | yes |
|  | CR | BM | <5 | negative | yes | yes | yes | yes | yes |
|  | Rel | BM | 50 | positive | yes | yes | yes | yes | yes |
| 03 | D | PB | 82 | positive | yes | yes | yes | yes | fail |
|  | CR | BM | 2-3 | negative | yes | yes | yes | yes | fail |
|  | Rel | BM | 50 | positive | yes | yes | yes | yes | fail |
| 04 | D | PB | 70 | positive | yes | yes | yes | yes | yes |
|  | CR | BM | 2 | negative | yes | yes | yes | yes | yes |
|  | Rel | BM | 17 | positive | yes | no | yes | yes | no |
| 05 | D | BM | 60 | positive | yes | yes | yes | yes | yes |
|  | CR | BM | <5 | positive | yes | yes | yes | yes | yes |
|  | Rel | PB | 59 | positive | yes | yes | yes | yes | yes |
| 06 | D | PB | 95 | positive | yes | yes | yes | yes | yes |
|  | CR | BM | <5 | negative | yes | yes | yes | yes | yes |
|  | Rel | BM | 15 | positive | no | no | no | no | no |
| 07 | D | BM | 90 | positive | yes | yes | yes | yes | yes |
|  | CR | BM | 0 | positive | no | no | no* | no* | no |
|  | Rel | BM | 80 | positive | yes | yes | yes | yes | fail |
| 08 | D | BM | 80 | positive | yes | yes | yes | yes | yes |
|  | CR | - | - | - | no | no | no* | no* | no |
|  | Rel | BM | 20-25 | positive | yes | no | yes | yes | no |
| 09 | D | PB | 40 | positive | yes | yes | yes | yes | yes |
|  | CR | BM | <2 | positive | yes | yes | yes | yes | yes |
|  | Rel | PB | 50 | positive | yes | no | yes | yes | no |

D: diagnosis, CR: complete remission, Rel: relapse, BM: bone marrow, PB: peripheral blood, WES: whole exome sequencing, scDNA-seq: single-cell DNA sequencing

*T cells from diagnosis were used as germline control

**Table S4:** Regions covered by the custom targeted sequencing panel.

| **Gene** | **Exons covered** |  | **Gene** | **Exons covered** |
| --- | --- | --- | --- | --- |
| *ASXL1* | full coding sequence |  | *KRAS* | full coding sequence |
| *ATM* | full coding sequence |  | *MPL* | Exon 10 |
| *BCOR* | full coding sequence |  | *MYD88* | full coding sequence |
| *BCORL1* | full coding sequence |  | *NF1* | Exons 28-38 |
| *BRAF* | Exon 15 |  | *NOTCH1* | Exons 26, 27, 34 |
| *BRCC3* | full coding sequence |  | *NPM1* | Exon 11 |
| *CALR* | Exons 8-9 |  | *NRAS* | full coding sequence |
| *CBL* | full coding sequence |  | *PHF6* | Exons 3-5, 7-8 |
| *CEBPA* | full coding sequence |  | *PPM1D* | full coding sequence |
| *CHEK2* | full coding sequence |  | *PTPN11* | full coding sequence |
| *CSF3R* | Exons 14, 17 |  | *RAD21* | full coding sequence |
| *DNMT3A* | full coding sequence |  | *RUNX1* | full coding sequence |
| *ETV6* | full coding sequence |  | *SETBP1* | Exons 4-9 |
| *EZH2* | full coding sequence |  | *SF3B1* | full coding sequence |
| *FLT3* | Exons 6, 14, 15, 20 |  | *SRSF2* | full coding sequence |
| *GATA1* | Exon 2 |  | *STAG2* | full coding sequence |
| *GATA2* | full coding sequence |  | *STAT3* | full coding sequence |
| *GNAS* | full coding sequence |  | *TET2* | full coding sequence |
| *GNB1* | full coding sequence |  | *TP53* | full coding sequence |
| *IDH1* | full coding sequence |  | *U2AF1* | full coding sequence |
| *IDH2* | full coding sequence |  | *WT1* | full coding sequence |
| *JAK2* | full coding sequence |  | *XPO1* | Exon 14 |
| *KIT* | Exons 8-11, 17 |  |  |  |

**Table S5:** Breakpoints of fusion genes detected with nanopore sequencing

| **Pat** | **Fusion type** | **5' Gene** | **5' Break** | **5' Position** | **3' Gene** | **3' Break** | **3' Position** | **Reads** |
| --- | --- | --- | --- | --- | --- | --- | --- | --- |
| 01 | intron-intron | *RUNX1* | intron 5-6 | 21:36217048 | *RUNX1T1* | intron 1-2 | 8:93081539 | 4/14 |
| 01 | intron-intron | *RUNX1T1* | intron 1-2 | 8:93081575 | *RUNX1* | intron 5-6 | 21:36217038 | 2/12 |
| 02 | intron-exon | *CBFB* | intron 5-6 | 16:67123598 | *MYH11* | exon 33-34 | 16:15815316 | 3/8 |
| 03 | intron-intron | *CBFB* | intron 5-6 | 16:67130917 | *MYH11* | intron 28-29 | 16:15826021 | 5/9 |
| 04 | intron-exon | *CBFB* | intron 5-6 | 16:67129470 | *MYH11* | exon 33-34 | 16:15815334 | 5/18 |
| 04 | exon-intron | *MYH11* | exon 33-34 | 16:15815335 | *CBFB* | intron 5-6 | 16:67129474 | 7/20 |
| 05 | intron-exon | *CBFB* | intron 5-6 | 16:67129212 | *MYH11* | exon 33-34 | 16:15815414 | 3/10 |
| 05 | exon-intron | *MYH11* | exon 33-34 | 16:15815425 | *CBFB* | intron 5-6 | 16:67129214 | 2/9 |
| 06 | intron-exon | *CBFB* | intron 5-6 | 16:67125015 | *MYH11* | exon 33-34 | 16:15815356 | 3/6 |
| 06 | exon-intron | *MYH11* | exon 33-34 | 16:15815357 | *CBFB* | intron 5-6 | 16:67125018 | 2/5 |
| 07 | intron-exon | *CBFB* | intron 5-6 | 16:67123190 | *MYH11* | exon 33-34 | 16:15815338 | 2/9 |
| 08 | intron-intron | *MYH11* | intron 33-34 | 16:15815187 | *CBFB* | intron 5-6 | 16:67116380 | 4/7 |
| 08 | intron-intron | *CBFB* | intron 5-6 | 16:67116362 | *MYH11* | intron 33-34 | 16:15815187 | 3/6 |
| 09 | intron-intron | *RUNX1* | intron 5-6 | 21:36227306 | *RUNX1T1* | intron 1-2 | 8:93053289 | 3/15 |

**Table S6:** Variants identified via bulk sequencing (WES and targeted sequencing) and single-cell DNA sequencing.

*See attached excel file*

**Table S7:** Custom targeted panels for MissionBio Tapestri single-cell DNA sequencing. CO-413 includes amplicons for patients 02, 04, 05 and 08; CO-414 includes amplicons for patients 01, 03 and 06; and CO-415 includes amplicons for patients 07 and 09.

*See attached excel file*

**Table S8:** Reference and alternative read counts for known variants in single-cell DNA sequencing samples used as an input for inferring tumor phylogenies. Cells are represented as columns, with cell barcodes as headers, and variants are listed in rows. Each variant in each cell is annotated as following: REF:ALT:GT. REF = reference allele counts, ALT = alternative/variant allele counts, GT = genotype. Genotype value, which is 0 for wild-type, 1 for heterozygous, 2 for homozygous and 3 for missing. This value is ignored for inferring tumor phylogenies.

*See attached excel file*

**Table S9:** Single-cell DNA sequencing parameters for every sample investigated. Panel uniformity: Percentage of amplicons that have mean reads to the amplicon above 0.2 * mean reads per amplicon per cell.

| **Sample ID** | **Time-point** | **Panel** | **No of Amplicons** | **Reads [x10^6^]** | **No of Cells** | **Mean reads/ cell/ amplicon** | **Panel Uniformity** |
| --- | --- | --- | --- | --- | --- | --- | --- |
| 01A | D | CO414 | 201 | 191 | 3337 | 57 | 90.05% |
| 01B | CR | CO414 | 201 | 391 | 2818 | 149 | 90.55% |
| 01C | Rel | CO414 | 201 | 184 | 4574 | 50 | 90.05% |
| 02A | D | CO413 | 180 | 251 | 7540 | 35 | 81.67% |
| 02B | CR | CO413 | 180 | 242 | 4103 | 83 | 84.44% |
| 02C | Rel | CO413 | 180 | 353 | 4125 | 142 | 84.44% |
| 03A | D | CO414 | 201 | 213 | 5287 | 60 | 90.55% |
| 03B | CR | CO414 | 201 | 330 | 4227 | 103 | 92.04% |
| 03C | Rel | CO414 | 201 | 187 | 4468 | 54 | 92.04% |
| 04A | D | CO413 | 180 | 346 | 1637 | 203 | 79.44% |
| 04B | CR | CO413 | 180 | 341 | 5333 | 107 | 83.33% |
| 05A | D | CO413 | 180 | 430 | 5526 | 128 | 87.22% |
| 05B | CR | CO413 | 180 | 289 | 2351 | 201 | 83.89% |
| 05C | Rel | CO413 | 180 | 250 | 2665 | 142 | 82.78% |
| 06A | D | CO414 | 201 | 185 | 2687 | 79 | 89.55% |
| 06B | CR | CO414 | 201 | 293 | 2459 | 130 | 90.55% |
| 07A | D | CO415 | 201 | 290 | 5884 | 64 | 89.55% |
| 07C | Rel | CO415 | 201 | 183 | 711 | 384 | 87.06% |
| 08A | D | CO413 | 180 | 370 | 2094 | 267 | 83.89% |
| 09A | D | CO415 | 201 | 291 | 4777 | 88 | 87.06% |
| 09B | CR | CO415 | 201 | 237 | 2144 | 140 | 89.05% |

# Supplementary Figures

**Figure S1:** Number of variants detected by whole exome or error-corrected targeted bulk sequencing for each patient in the diagnosis (D) and relapse (Rel) sample. Variants that are shared between D and Rel are blue, variants unique to diagnosis are orange and variants unique to relapse are colored yellow. For patient 06 no relapse sample was available. The total number of somatic variants per patient are shown in brackets.

**
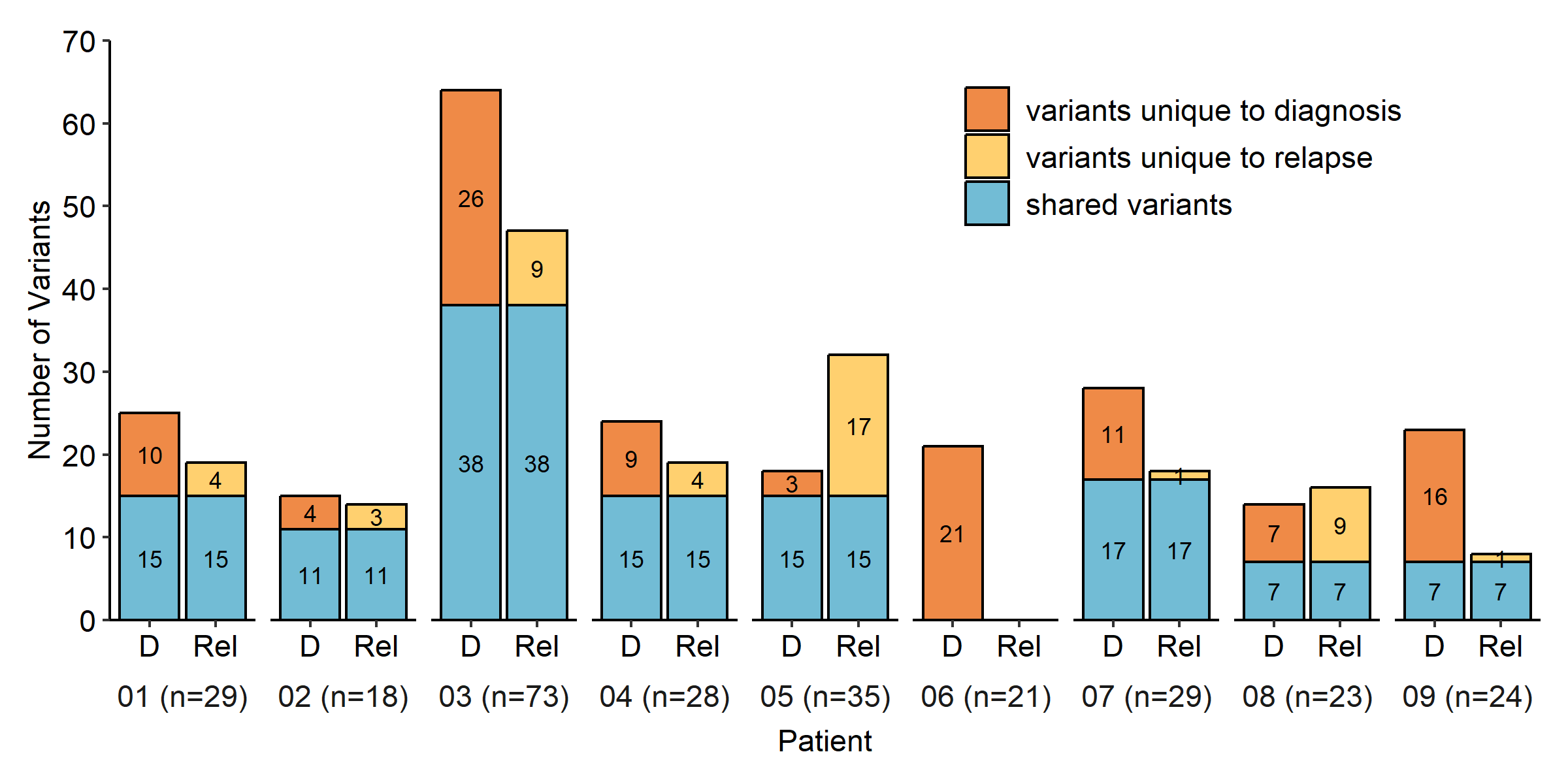
**

**Figure S2:** Mutational landscape of 9 core-binding factor (CBF) AML patients. Samples are grouped by timepoint (Diagnosis/Relapse). The top graph shows the number of variants per sample. Only the top mutated genes are shown (at least 2 patients are mutated).

**
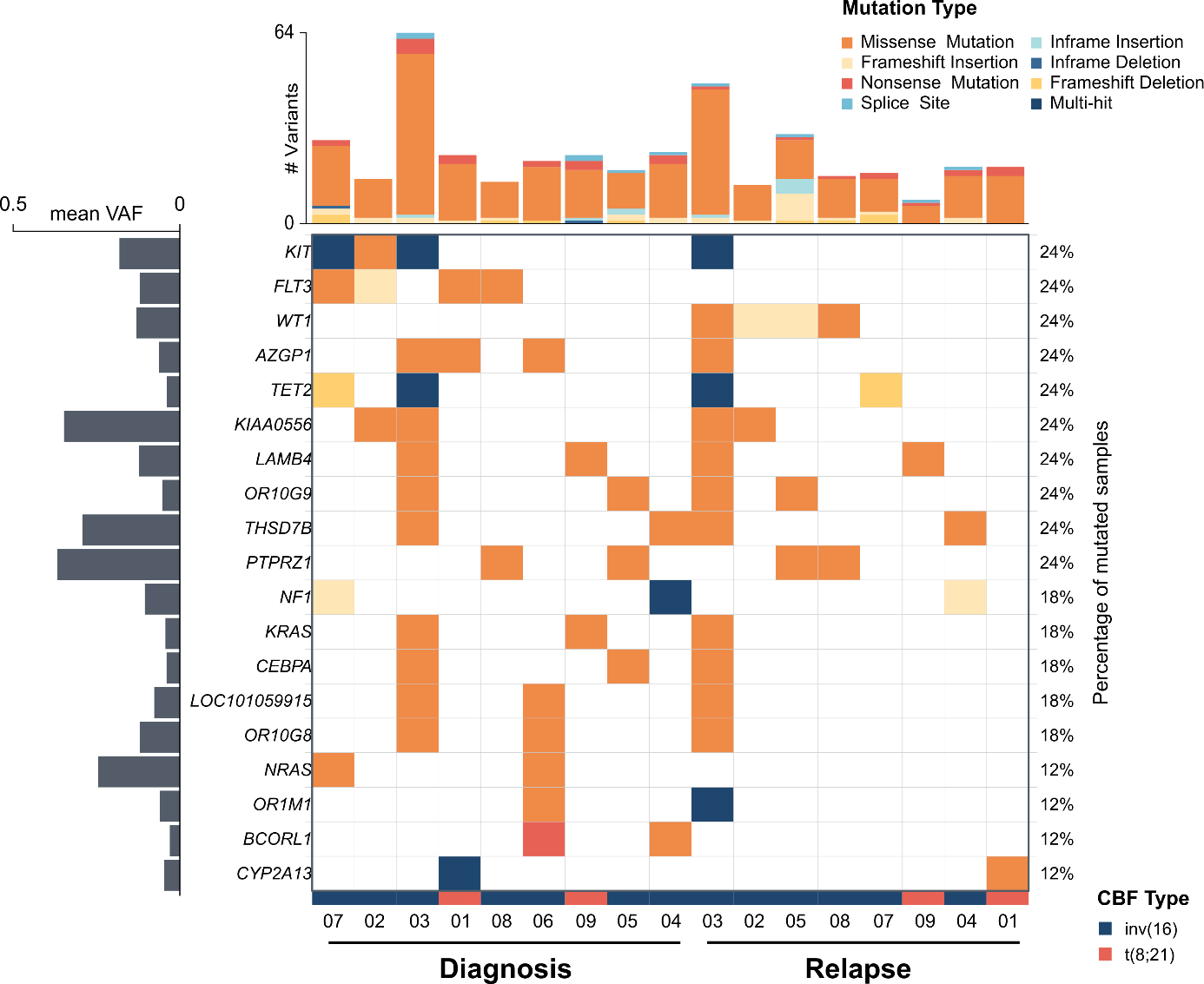
**


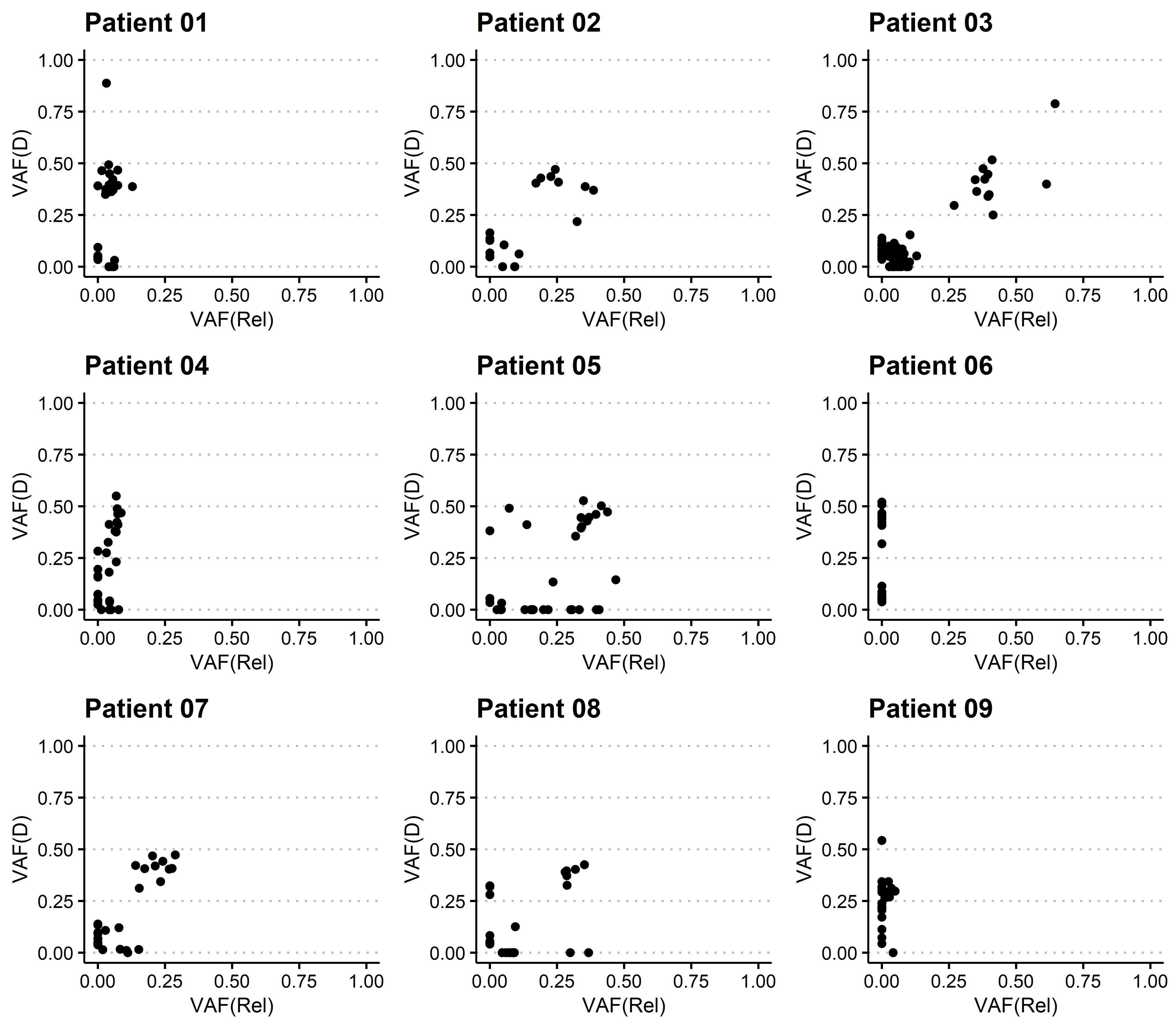
**Figure S3:** Variant allele frequency (VAF) of mutations identified in all patients with diagnosis (D) and relapse (Rel) samples via whole exome sequencing.

**Figure S4: A.** Density plot of variant allele frequencies (VAF) derived from bulk sequencing at D and Rel for all 405 somatic variants. Wilcoxon signed-rank test was used for statistical testing. **B** Box plot of blast counts in % in patient samples at diagnosis, complete remission and relapse. Differences of blast counts between timepoints were determined via Wilcoxon signed-rank test for paired samples.

**A**


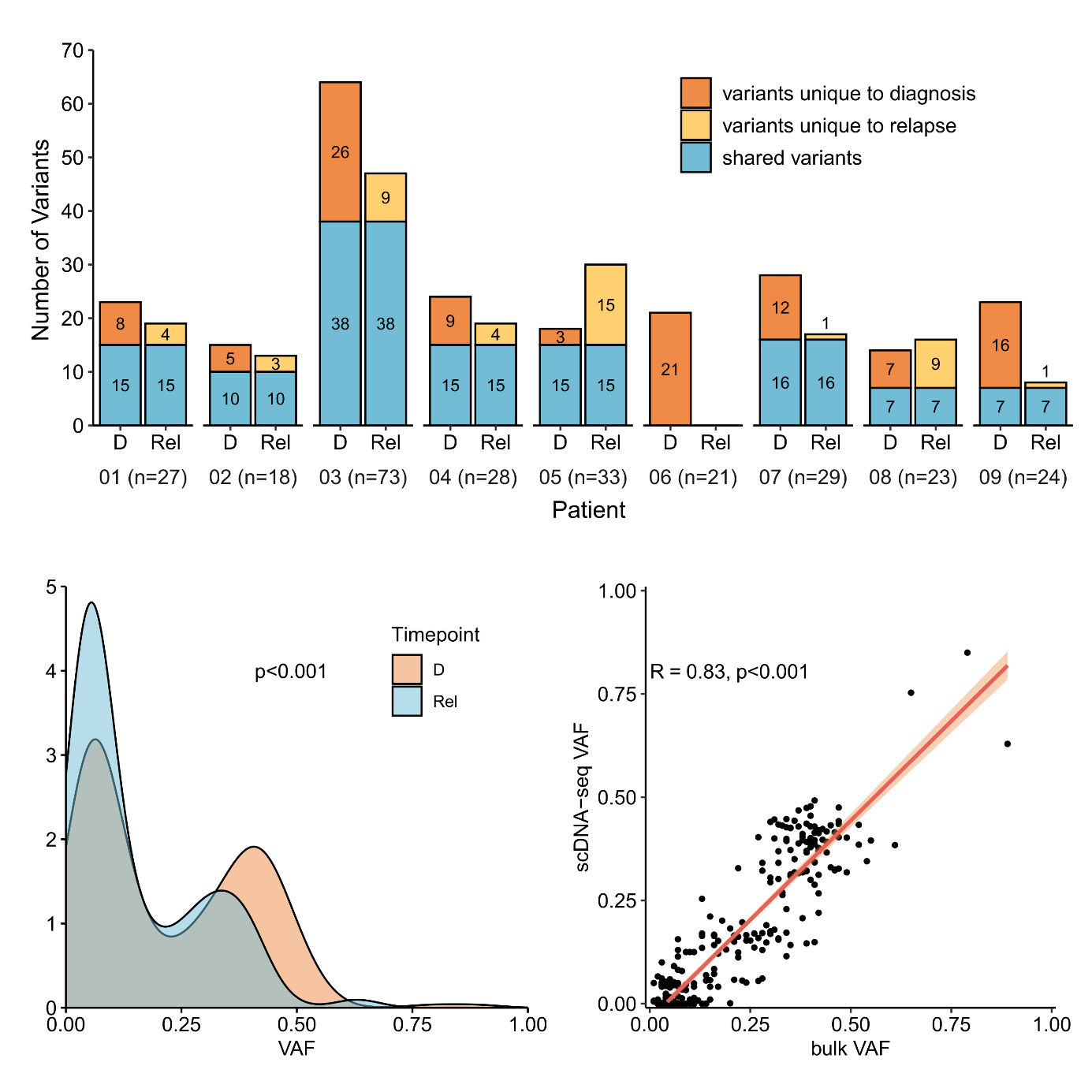

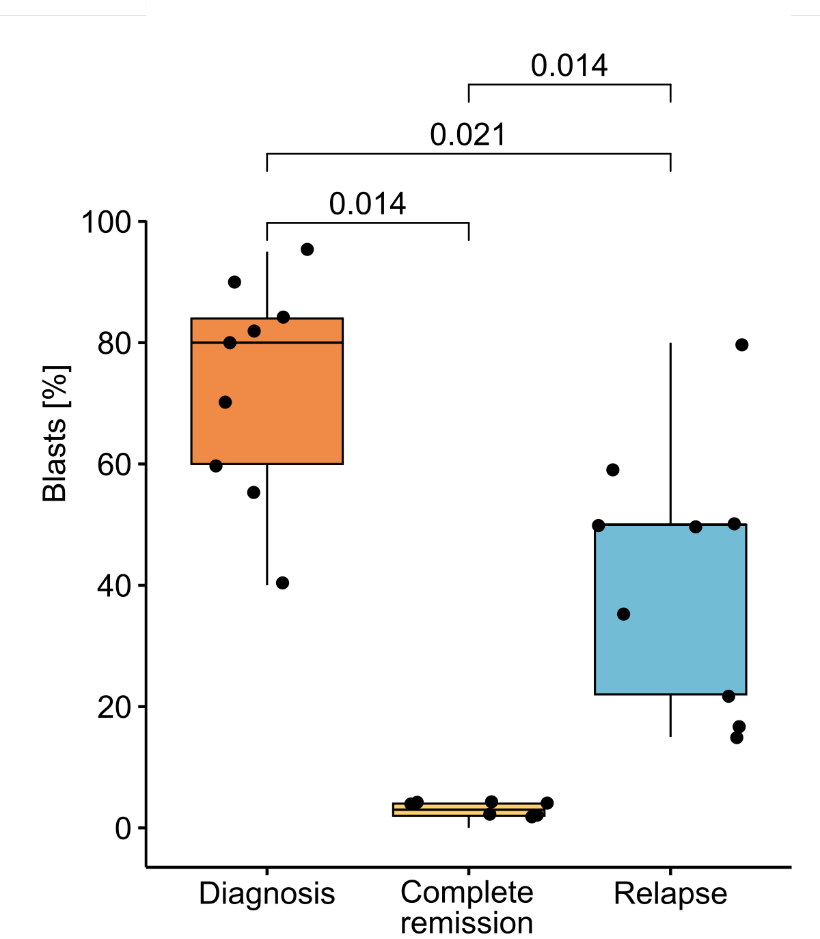


**B**

**Figure S5:** Genome plots showing somatic copy number alterations identified via whole exome sequencing for patients **A** 01, **B** 05, **C** 07, **D** 08 and **E** 09. Plots show purity, ploidy and copy-numbers of the major (yellow) and minor (blue) allele within every autosomal chromosome for each analyzed sample.

**
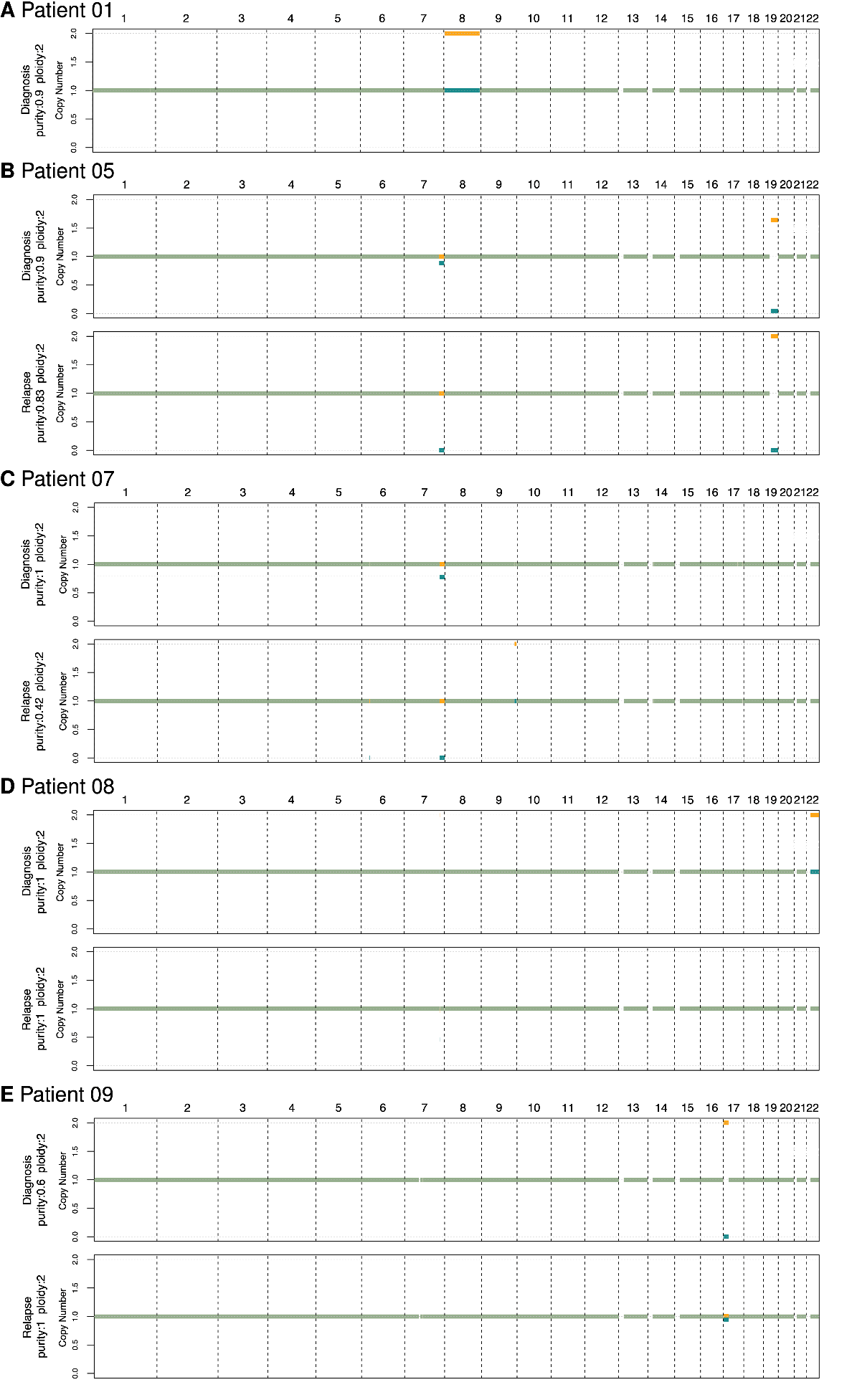
**

**
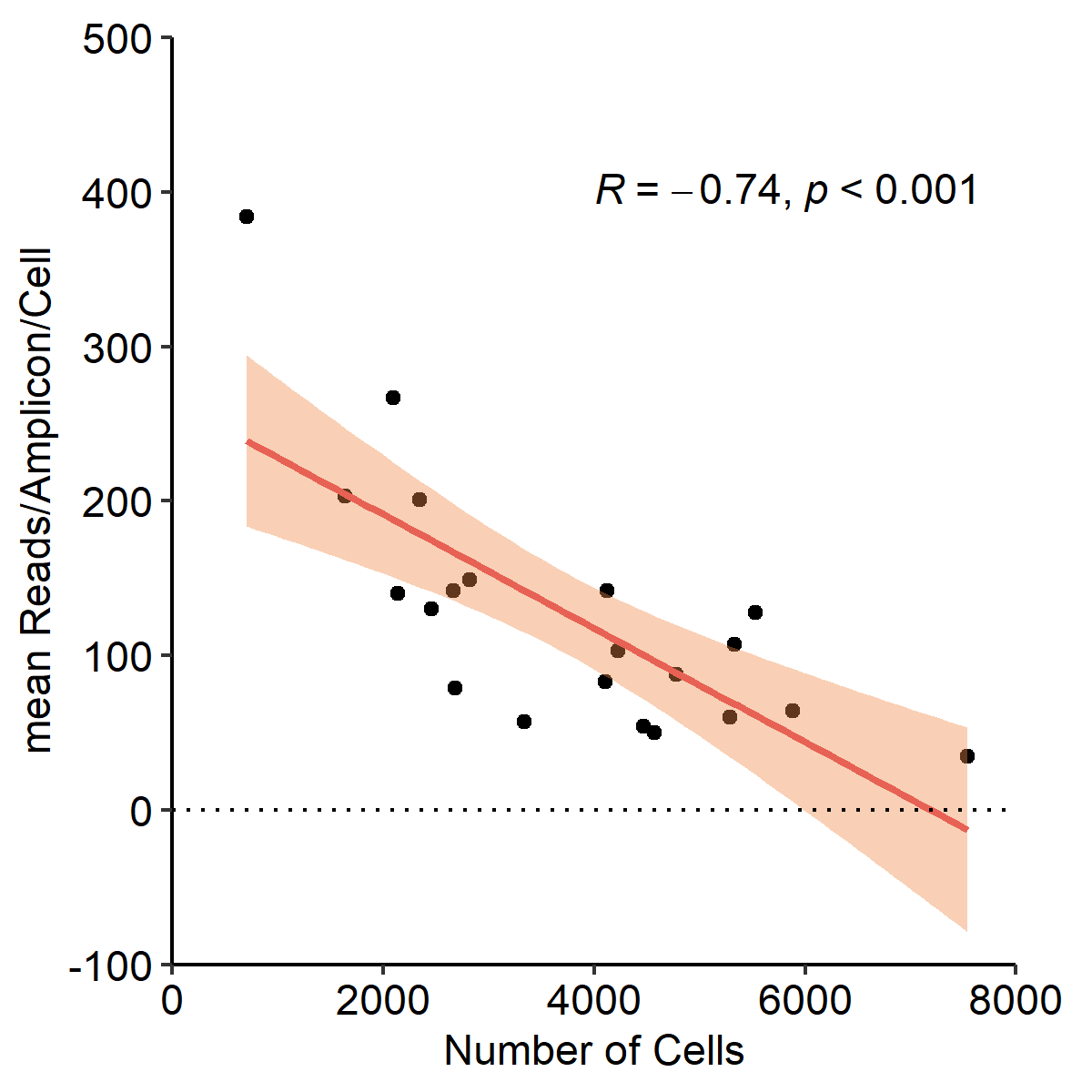
Figure S6:** Scatter plot showing correlation between mean reads per amplicon per cell and number of cells in samples sequenced with MissionBio Tapestri platform (n=21 samples). Correlation coefficient R and p-value were calculated using Spearman rank correlation.

**
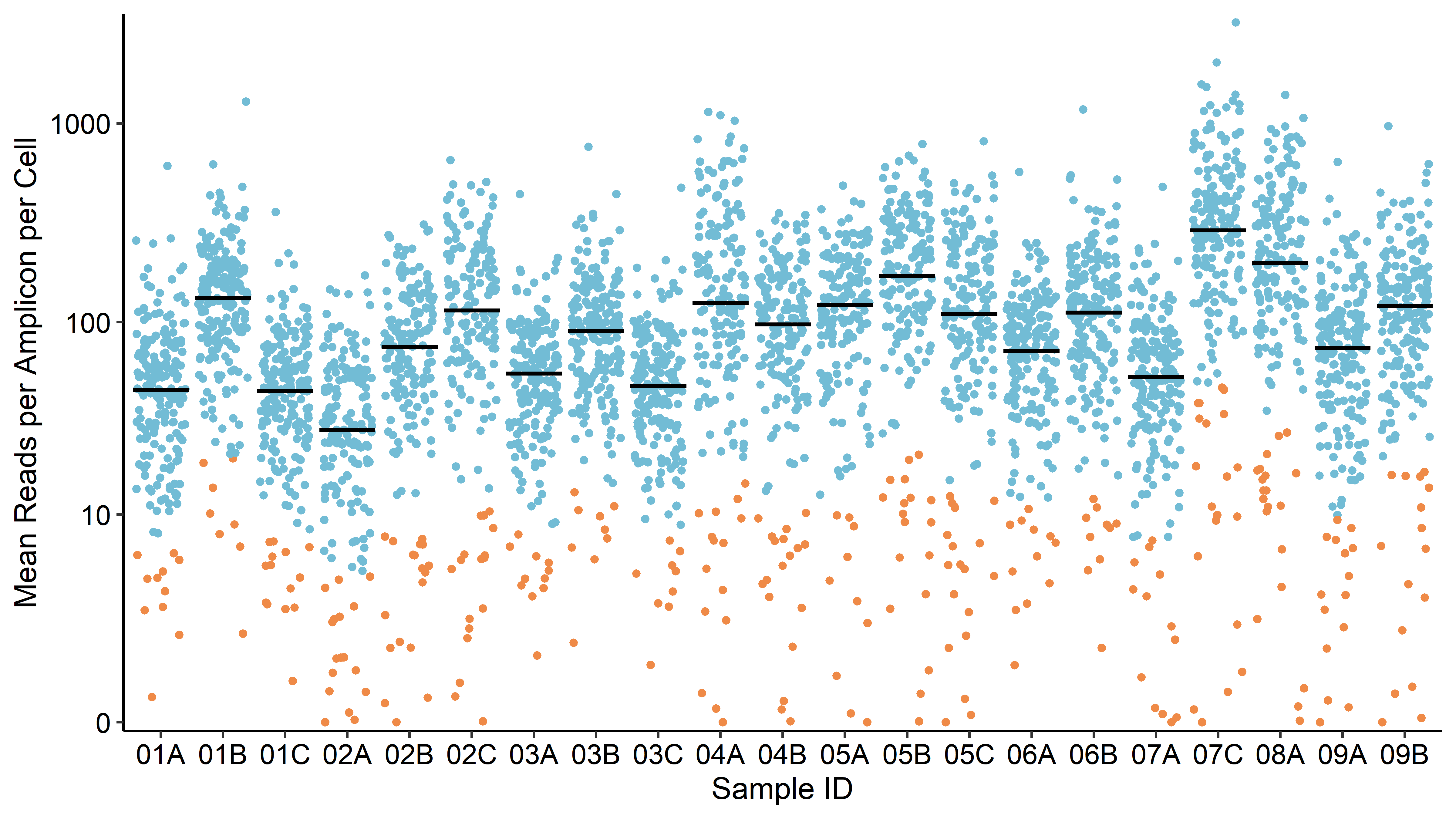
Figure S7:** Coverage statistics for samples sequenced on Mission Tapestri platform. Mean reads per amplicon per cell are shown for each amplicon and sample. Black bars indicate the median coverage per sample. Orange dots did not pass the Tapestri pipeline coverage threshold (<0.2* mean reads/amplicon/cell). The numbers below the plots indicate the patient number and the sample time, A: diagnosis, B: complete remission, C: relapse.

**
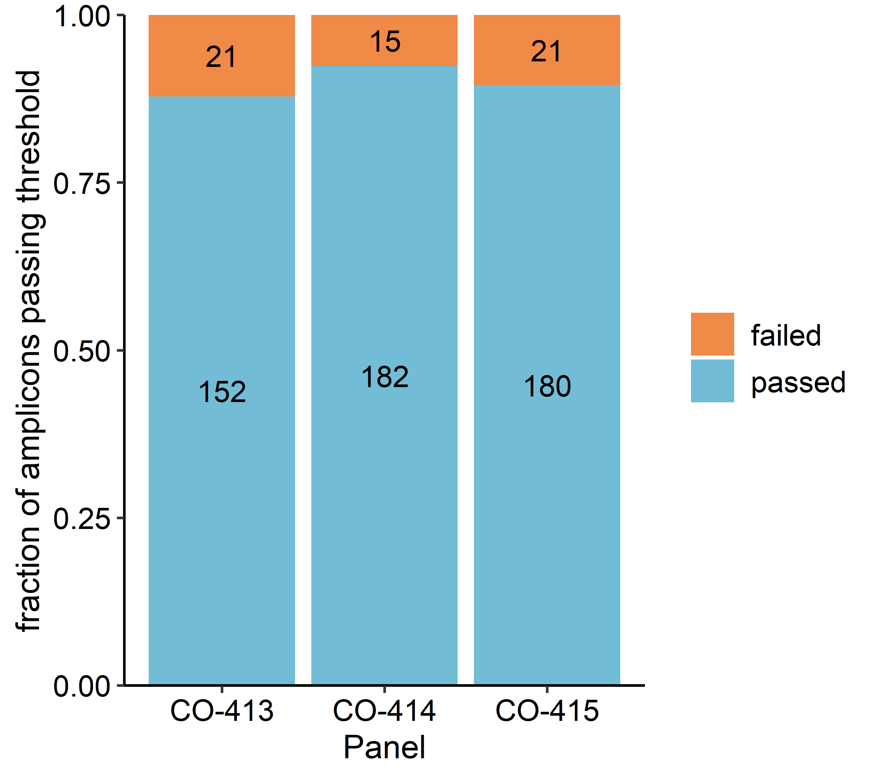
Figure S8:** Fractions of amplicons in each panel that passed coverage threshold (>0.2* mean reads/amplicon/cell).

**Figure S9:** Allelic dropout (ADO) rates of germline heterozygous variants in amplicons designed for copy-number analysis shown for each sample. Median of ADOs per sample are shown at the top. ADO = [cells with reference calls (REF) + cells with homozygous calls (HOM)] / [REF + HOM + cells with heterozygous calls (HET)]. The numbers below the plots indicate the patient number and the sample time, A: diagnosis, B: complete remission, C: relapse.

**
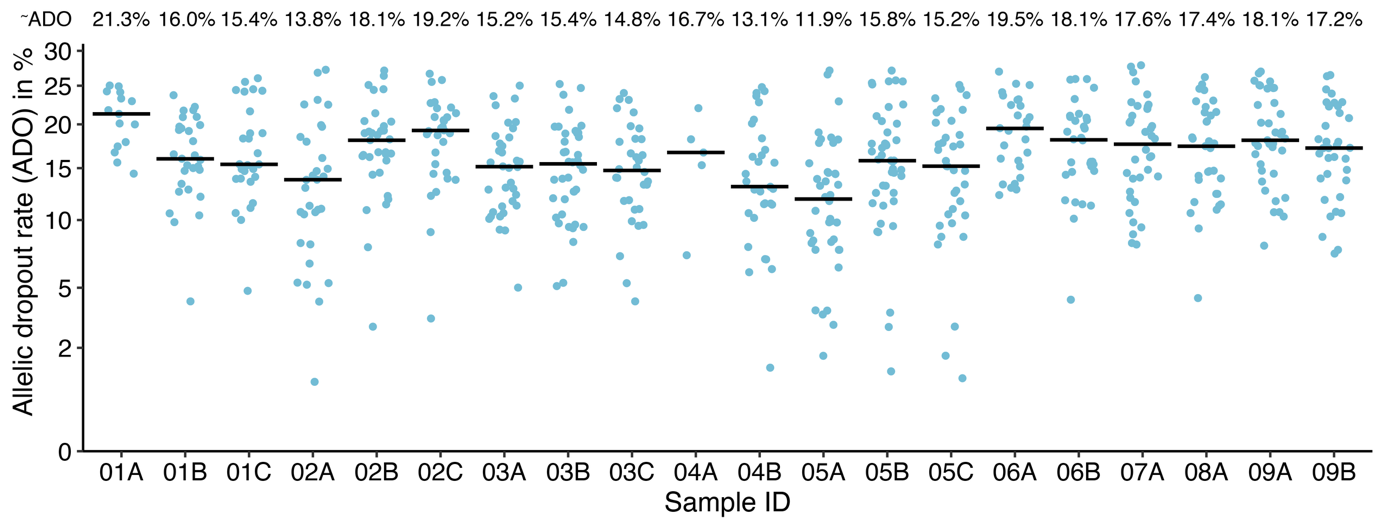
**

**Figure S10:** Fraction of variant overlap between bulk and single-cell sequencing. Variants from bulk that were also detected in scDNA-seq are colored blue (detected) and variants from bulk seq that were not found via scDNA-seq are colored orange (not detected). Numbers include variants from diagnosis and relapse samples per patient.

**
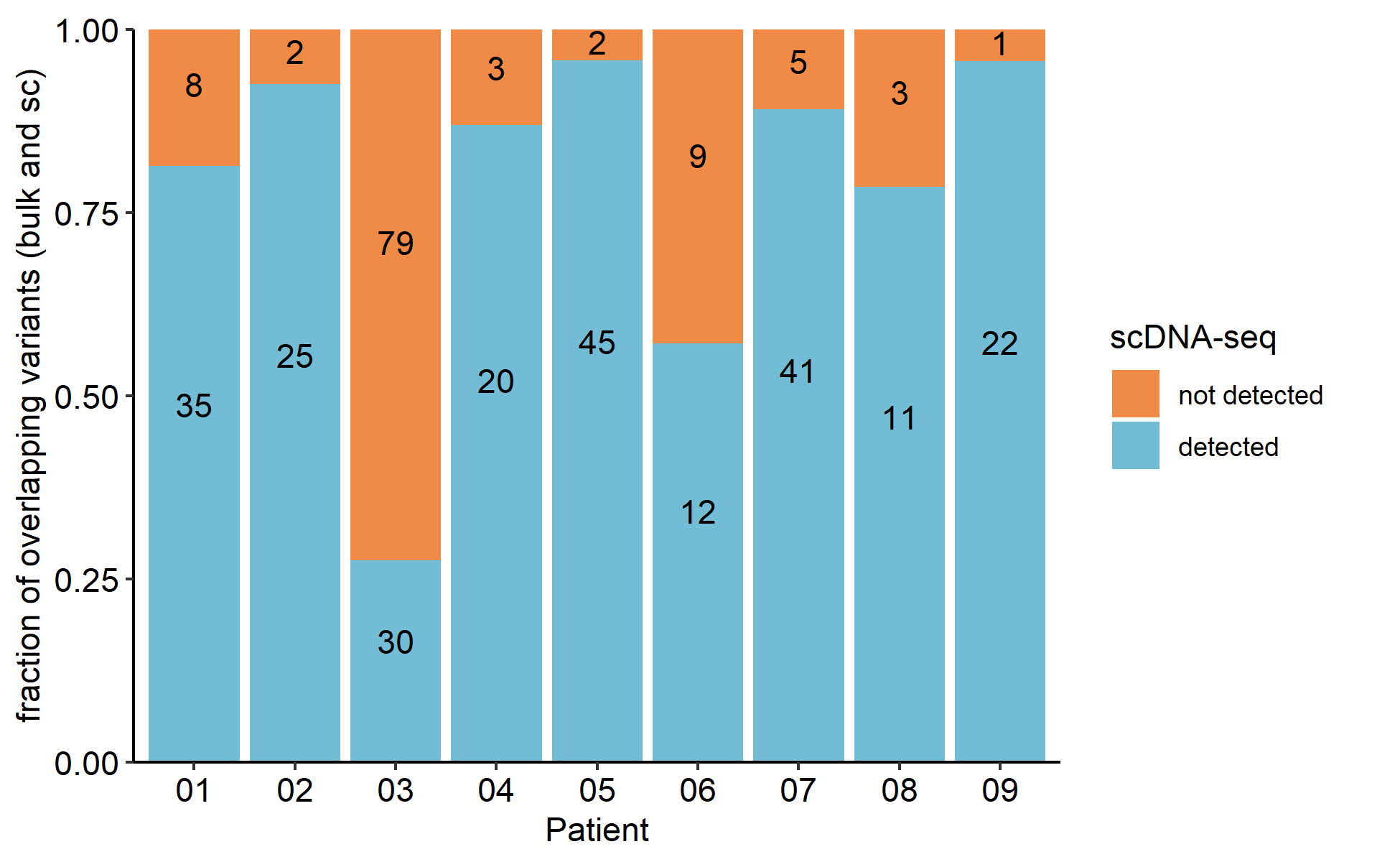
**

**Figure S11:** Scatter plots of single-cell DNA sequencing variant allele frequencies (scDNA-seq VAF) vs. VAF from bulk sequencing (bulk VAF). **A** Correlation coefficient (R) and p-value were calculated via Spearman rank correlation. The axes are square root-transformed for better visualization of smaller clones. **B** The axes are log10-transformed to visualize low VAF variants. Calculation of the correlation coefficient was omitted because of the log10 transformation. scDNA-seq VAF was inferred from single-cell genotyping: scDNA-seq VAF = (n(HOM cells) + n(HET cells)*0.5)/total number of cells.

**B**


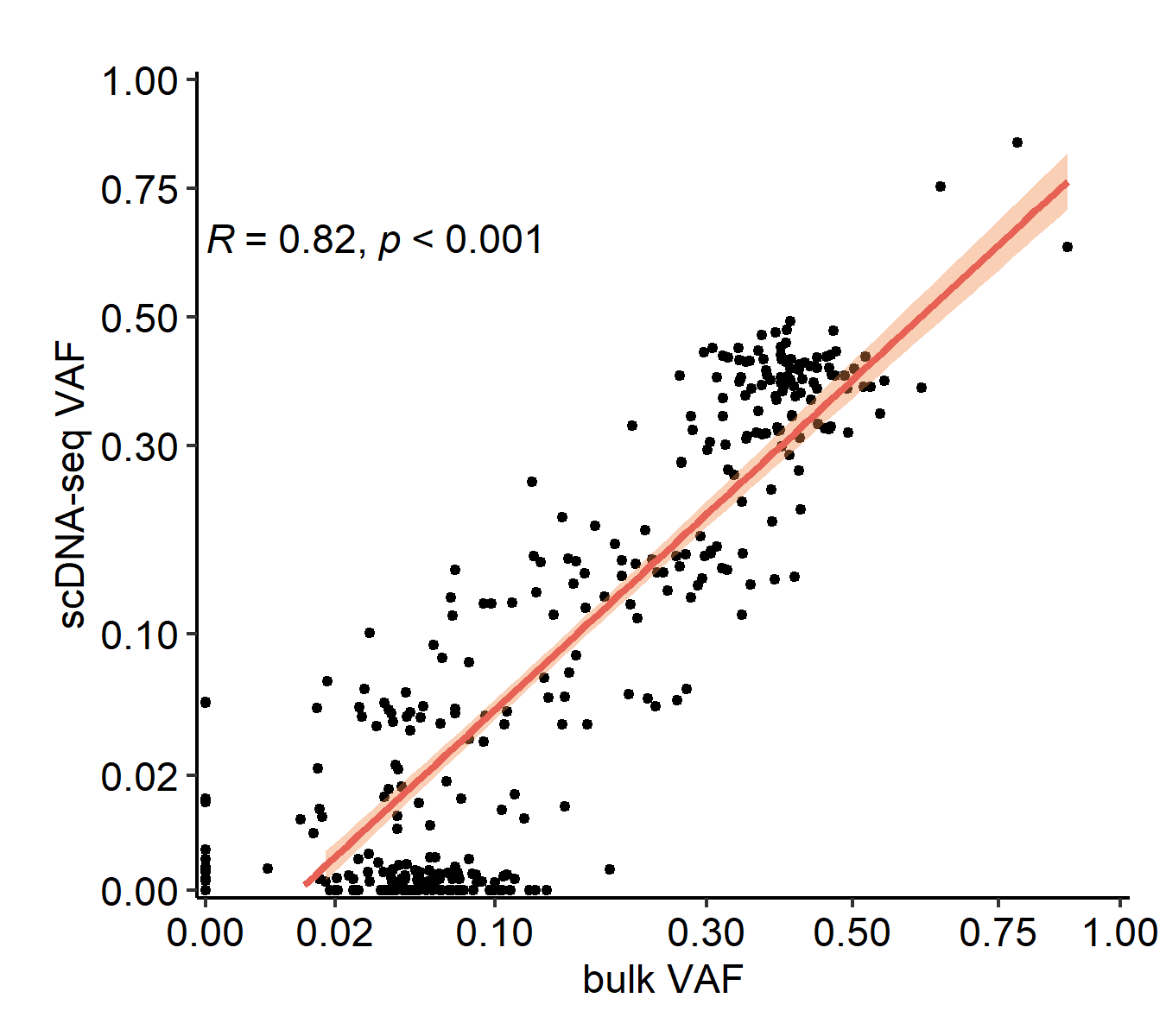

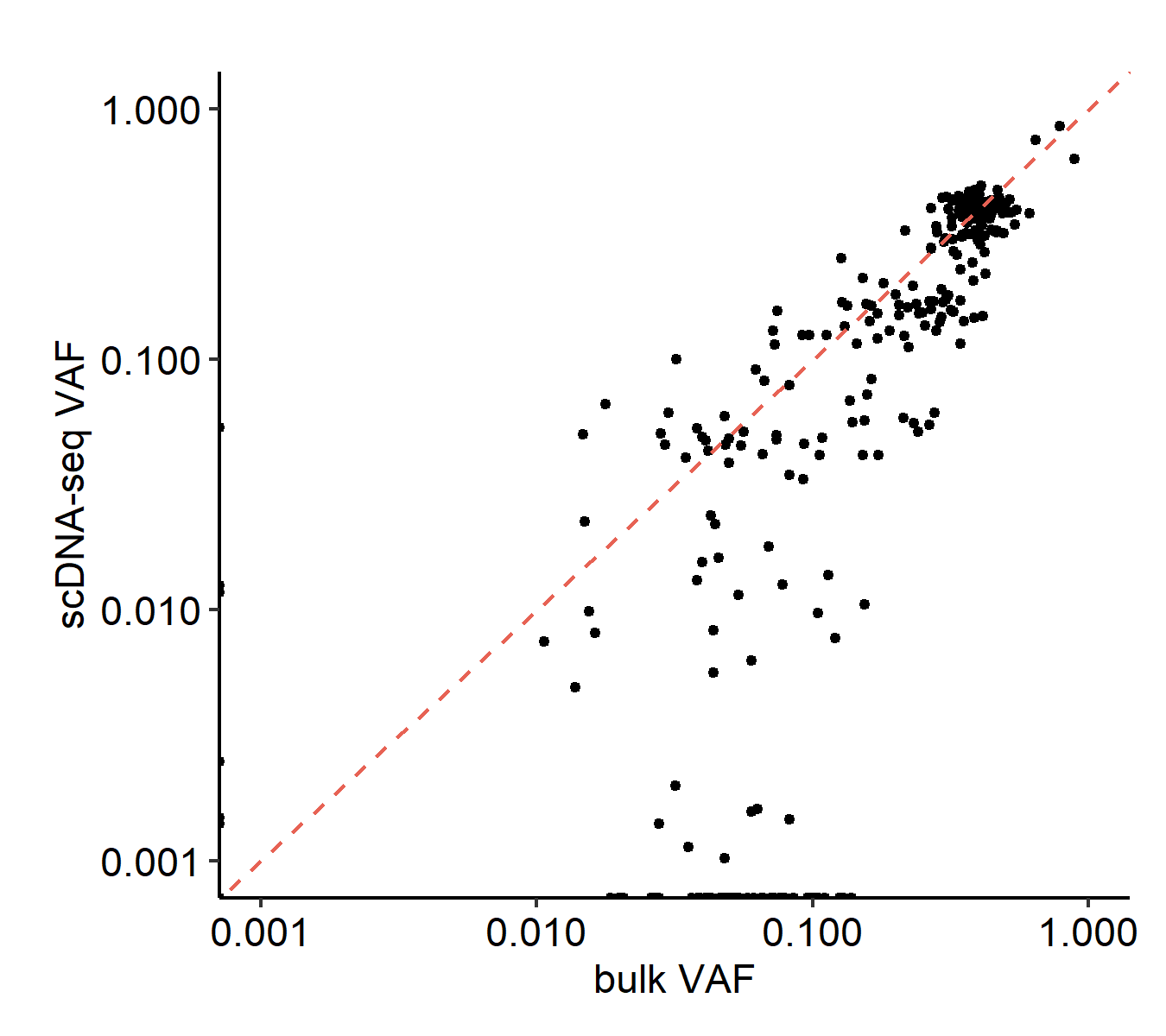


**A**


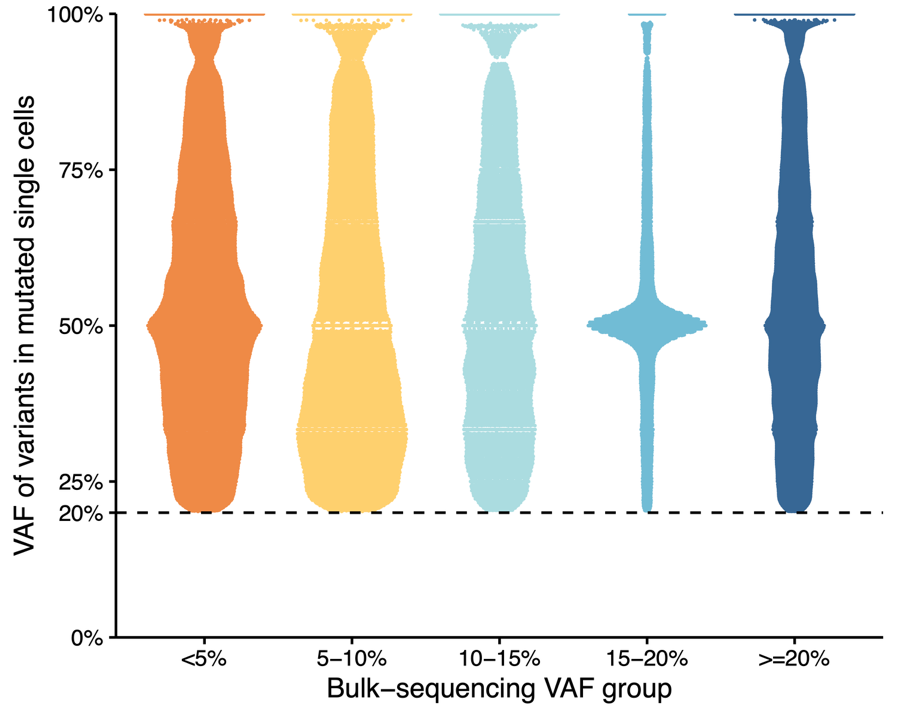
**Figure S12:** Bee swarm-style plot of variant allele frequencies (VAFs) of as mutated classified single cells grouped by the corresponding bulk sequencing VAF from the same sample. To be classified as mutated, a variant must meet specific criteria, including a VAF of at least 20% (represented by a dashed line).

**Figure S13:** Complete phylogenetic trees at diagnosis (D) as determined via scDNA-seq and modelling via COMPASS. Copy number alterations were integrated separately into the phylogenetic tree. All events used to infer the phylogenetic trees are shown here. CBF fusions and copy number alterations are shown in bold.

**
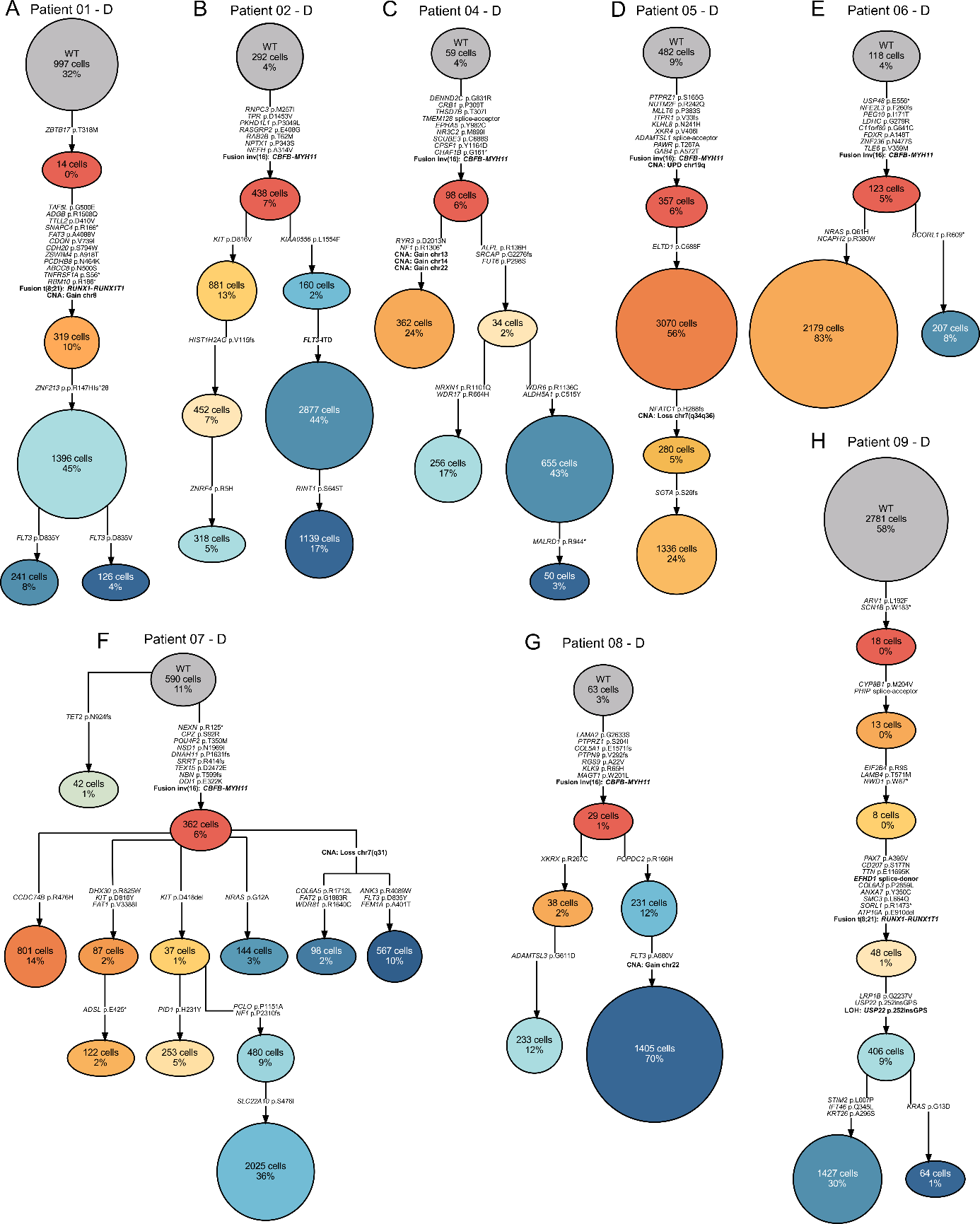
**

**Figure S14:** Combined phylogenetic trees from patients with diagnosis and relapse samples as determined via scDNA-seq and modelling via COMPASS. Copy number alterations were integrated separately into the phylogenetic tree. All events used to infer the phylogenetic trees are shown here. Variants and cell count from both timepoints were used together to model more accurate phylogenetic trees. Cell counts are indicated for diagnosis/relapse samples, respectively. CBF fusions and copy number alterations are shown in bold.


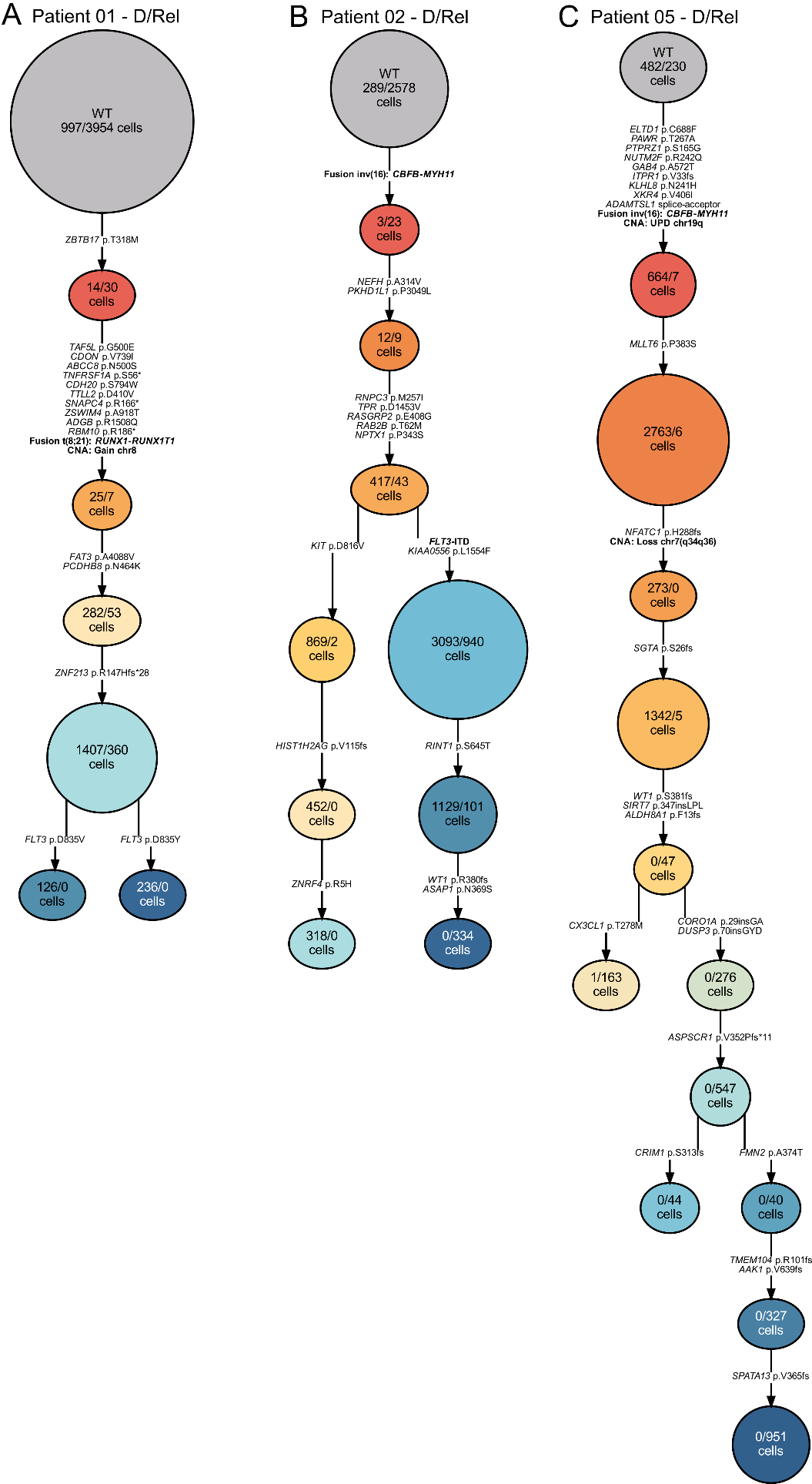


**Figure S15:** Number of unique cell barcodes that aligned to the sample specific fusion gene amplicons. Plots are grouped by panel: **A** CO-413, **B** CO-414, **C** CO-415. For each sample, reads for ≤3 cell barcodes mapped to fusion genes of a different patient, except for samples of patient 05 where reads of 491 (05A) and 70 (05C) barcodes mapped to the second gene fusion of patient 04. This might result from the *CBFB-MYH11* gene fusions of patient 04 and patient 05 having the 3’-breakpoints within 80bp (chr16:15815334 and chr16:15815414). The numbers above the plots indicate the patient number and the sample time, A: diagnosis, B: complete remission, C: relapse.

**
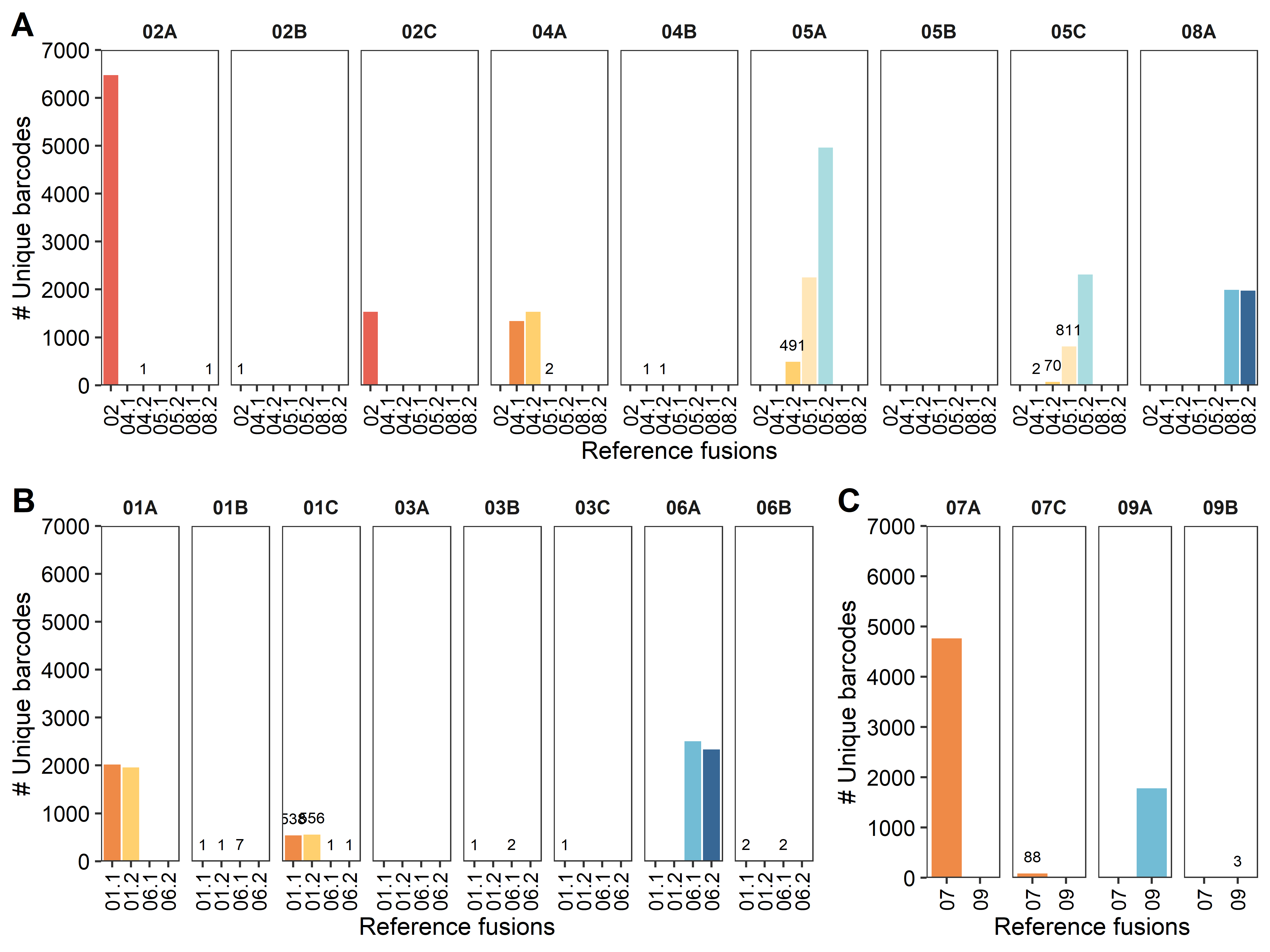
**

**Figure S16:** Heatmaps present single cells in columns and variants in rows at diagnosis (D) for patients **A** 01, **B** 02, **C** 04 and **D** 05. Variants are classified as wild-type (WT), heterozygous (HET), homozygous (HOM) or missing (MISS). Cells have been grouped by the inferred tumor phylogenies for each sample with cells flagged as doublets removed.

**
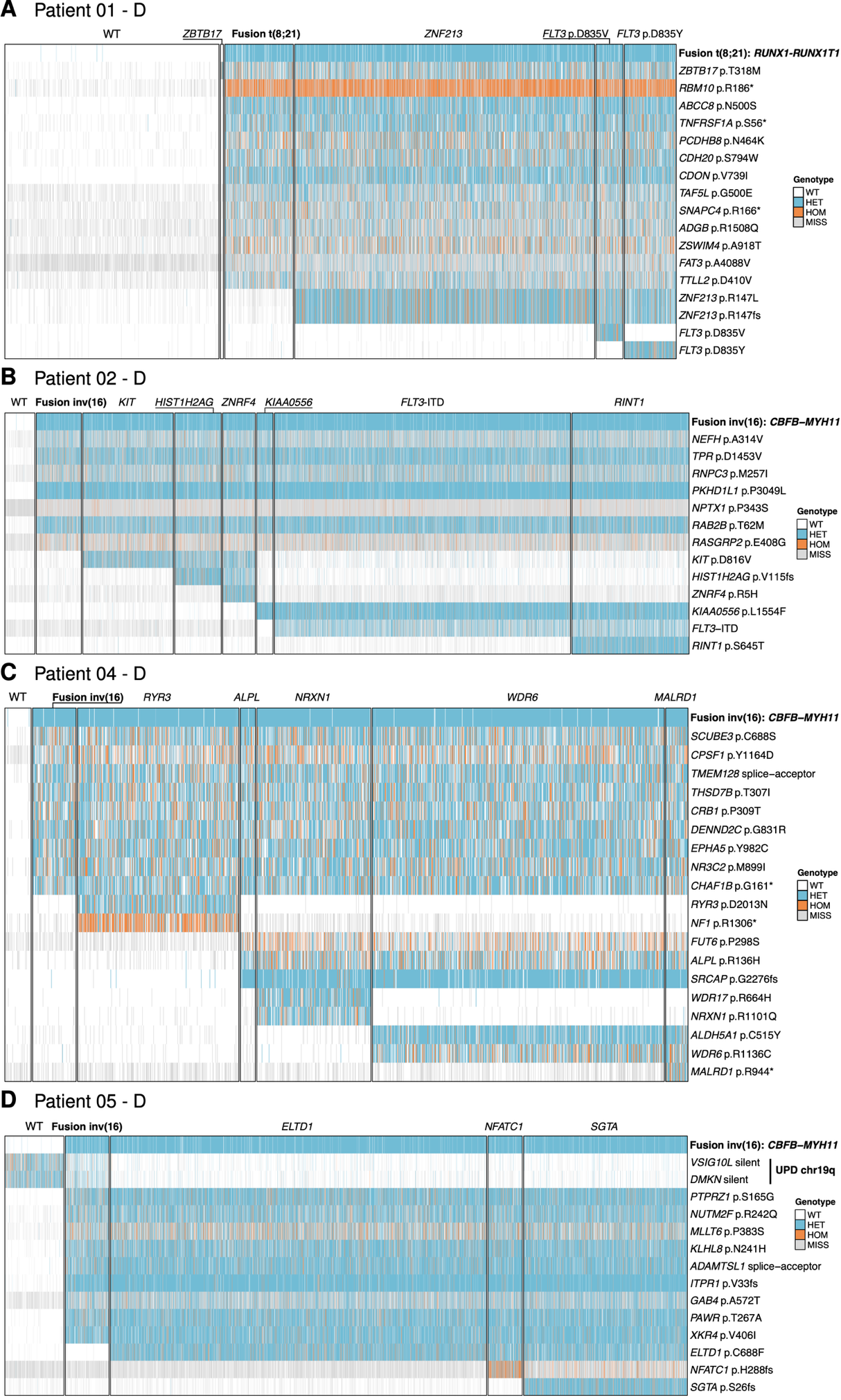
**

**Figure S17:** Heatmaps present single cells in columns and variants in rows at diagnosis (D) for patients **A** 06, **B** 07, **C** 08 and **D** 09. Variants are classified as wild-type (WT), heterozygous (HET), homozygous (HOM) or missing (MISS). Cells have been grouped by the inferred tumor phylogenies for each sample with cells flagged as doublets removed.

**
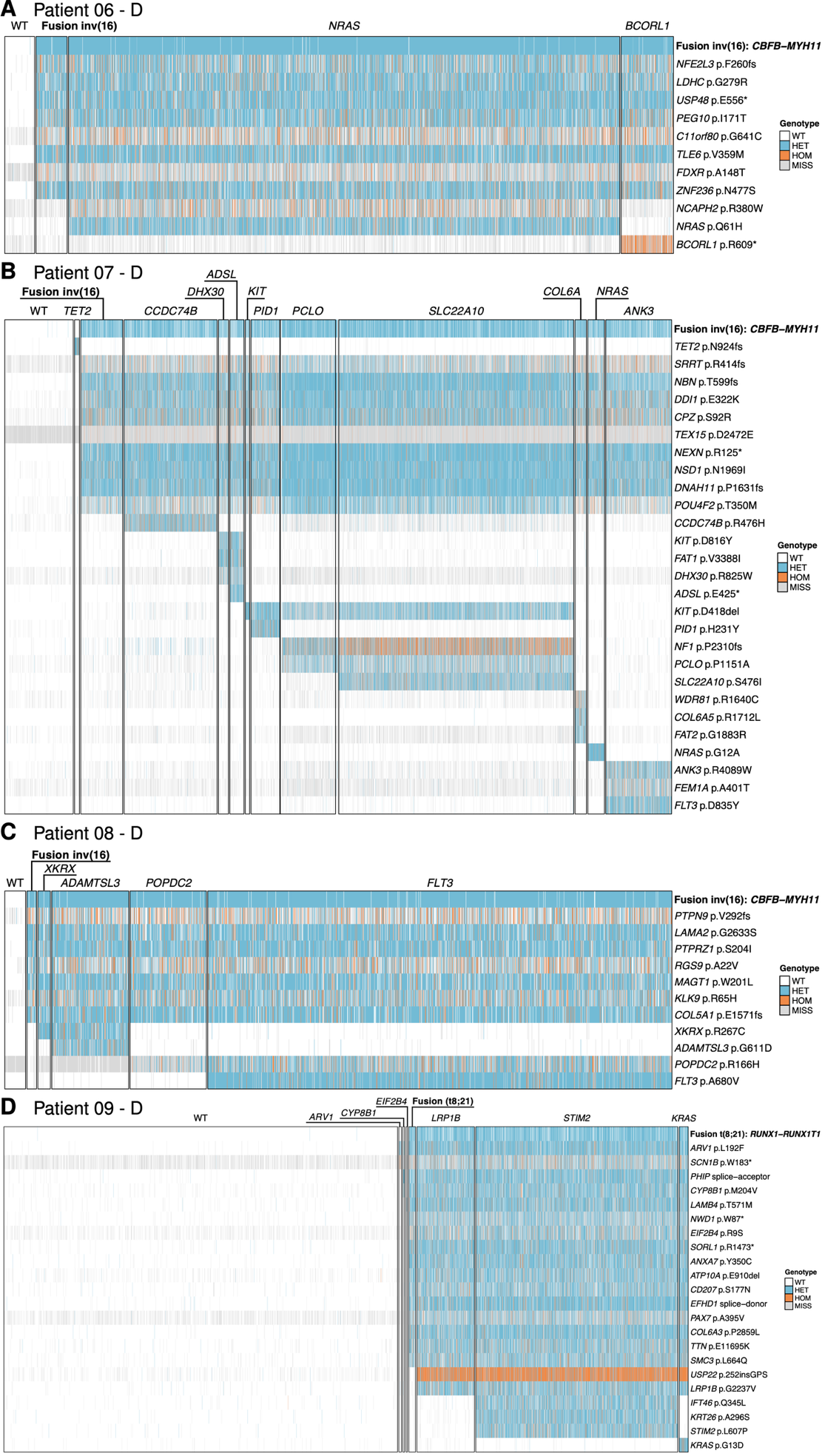
**

**Figure S18:** Heatmaps present single cells in columns and variants in rows for combined diagnosis (D) and relapse (Rel) samples of patient 01. Variants are classified as wild-type (WT), heterozygous (HET), homozygous (HOM) or missing (MISS). Cells have been grouped by the inferred tumor phylogenies for the combined samples with cells flagged as doublets removed. In **A** the heatmap shows cells from both samples and in **B** the cells are split by their origin.

**
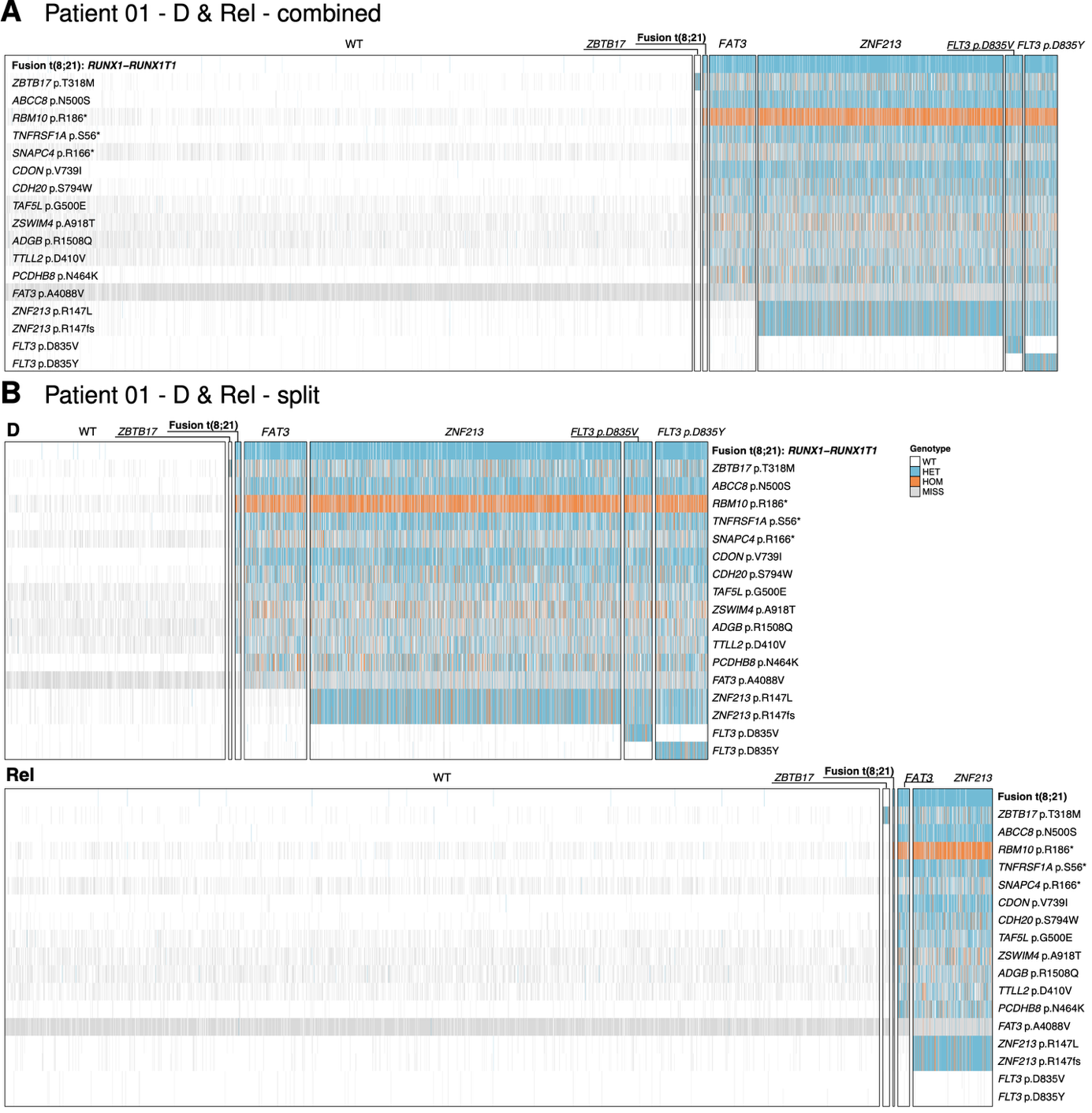
**

**Figure S19:** Heatmaps present single cells in columns and variants in rows for combined diagnosis (D) and relapse (Rel) samples of patient 02. Variants are classified as wild-type (WT), heterozygous (HET), homozygous (HOM) or missing (MISS). Cells have been grouped by the inferred tumor phylogenies for the combined samples with cells flagged as doublets removed. In **A** the heatmap shows cells from both samples and in **B** the cells are split by their origin.

**
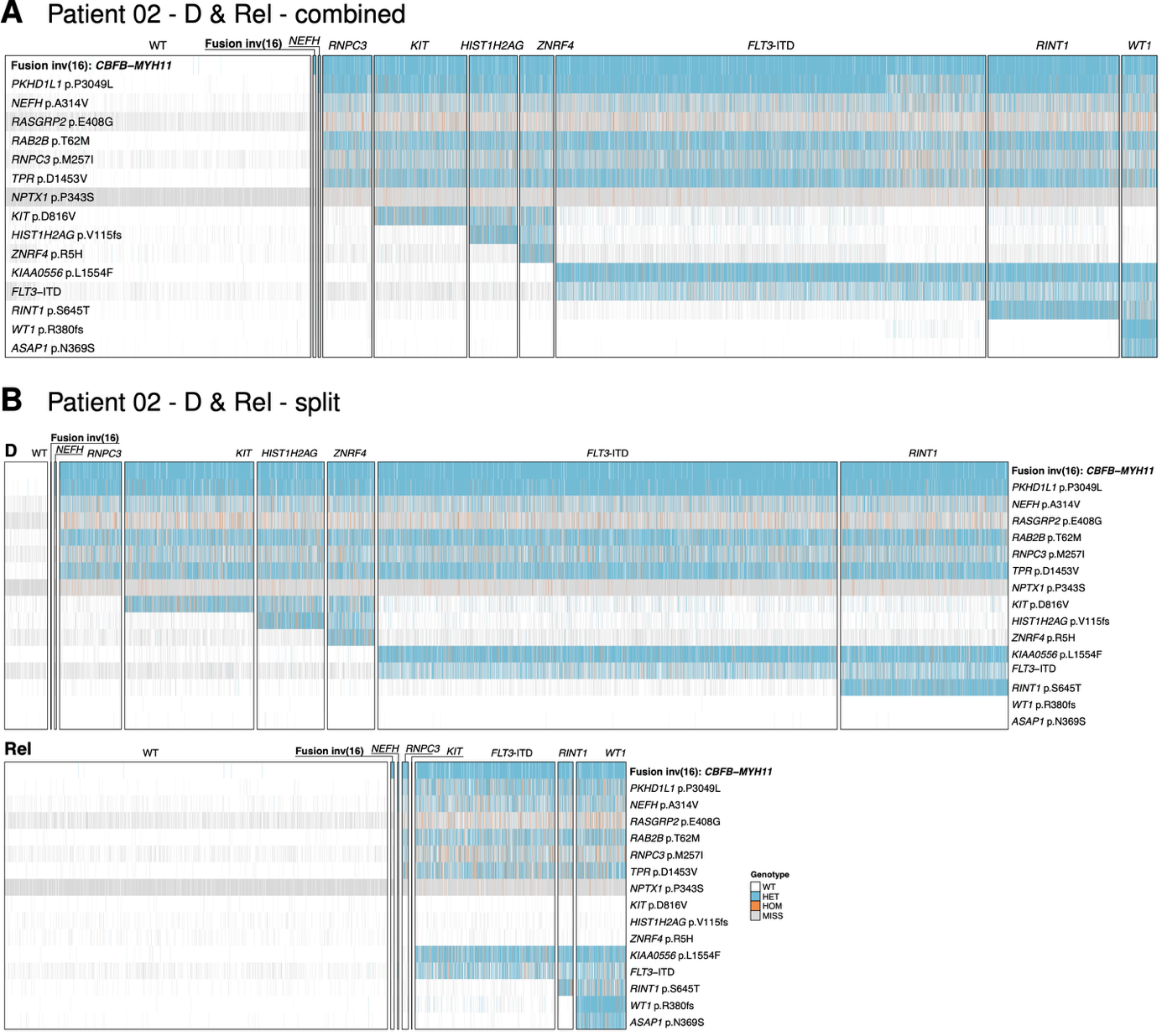
**

**Figure S20:** Heatmaps present single cells in columns and variants in rows for combined diagnosis (D) and relapse (Rel) samples of patient 05. Variants are classified as wild-type (WT), heterozygous (HET), homozygous (HOM) or missing (MISS). Cells have been grouped by the inferred tumor phylogenies for the combined samples with cells flagged as doublets removed. In **A** the heatmap shows cells from both samples and in **B** the cells are split by their origin.

**
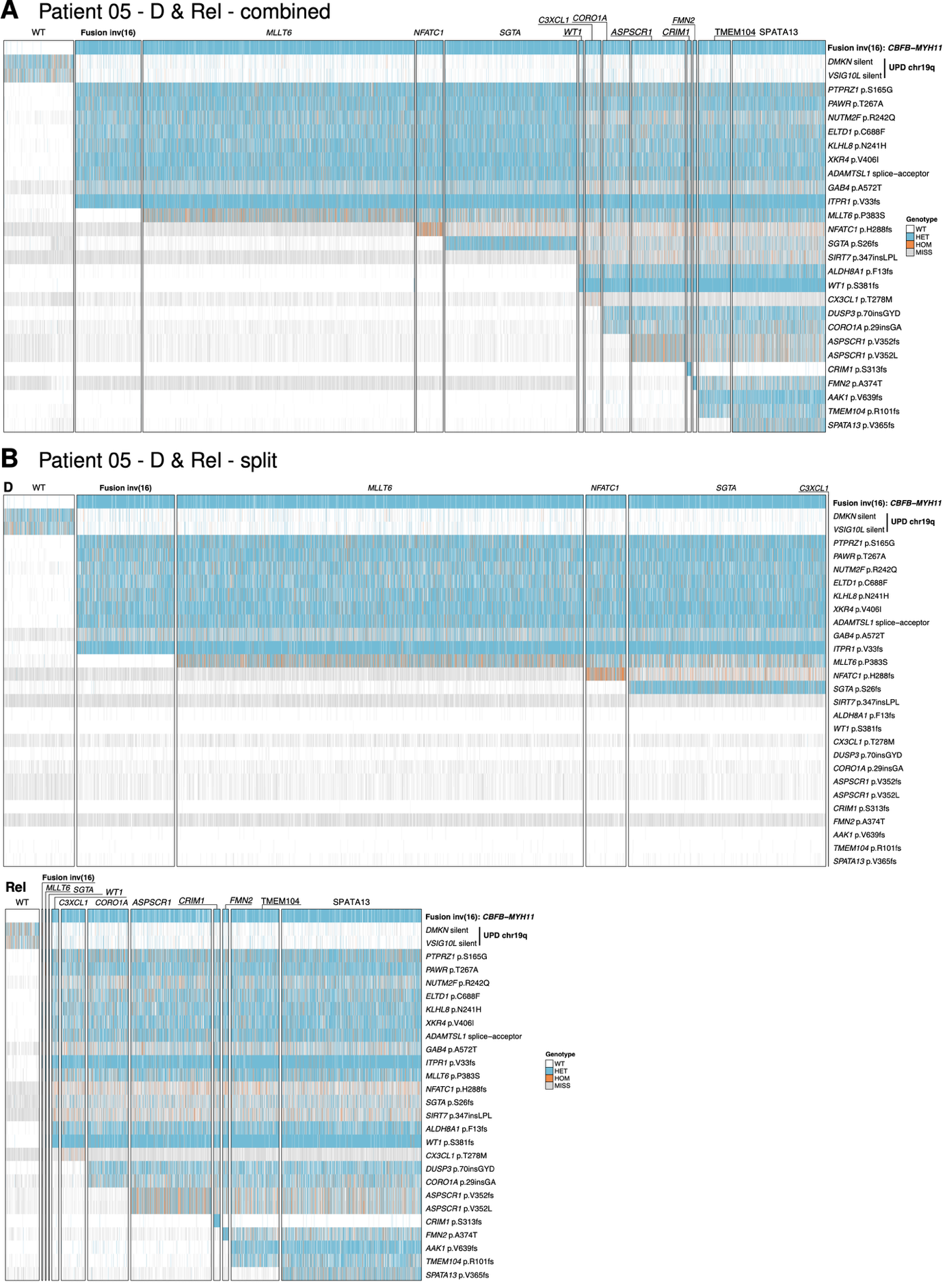
Figure S21:** Heatmaps present single cells in columns and variants in rows for inferred clones before and the t(8;21) fusion clone for **A** .combined diagnosis (D) and relapse (Rel) samples of patient 01 and **B** for the diagnosis sample of patient 09. Variants are classified as wild-type (WT), heterozygous (HET), homozygous (HOM) or missing (MISS). Cells have been grouped by the inferred tumor phylogenies for the combined samples with cells flagged as doublets removed.

**
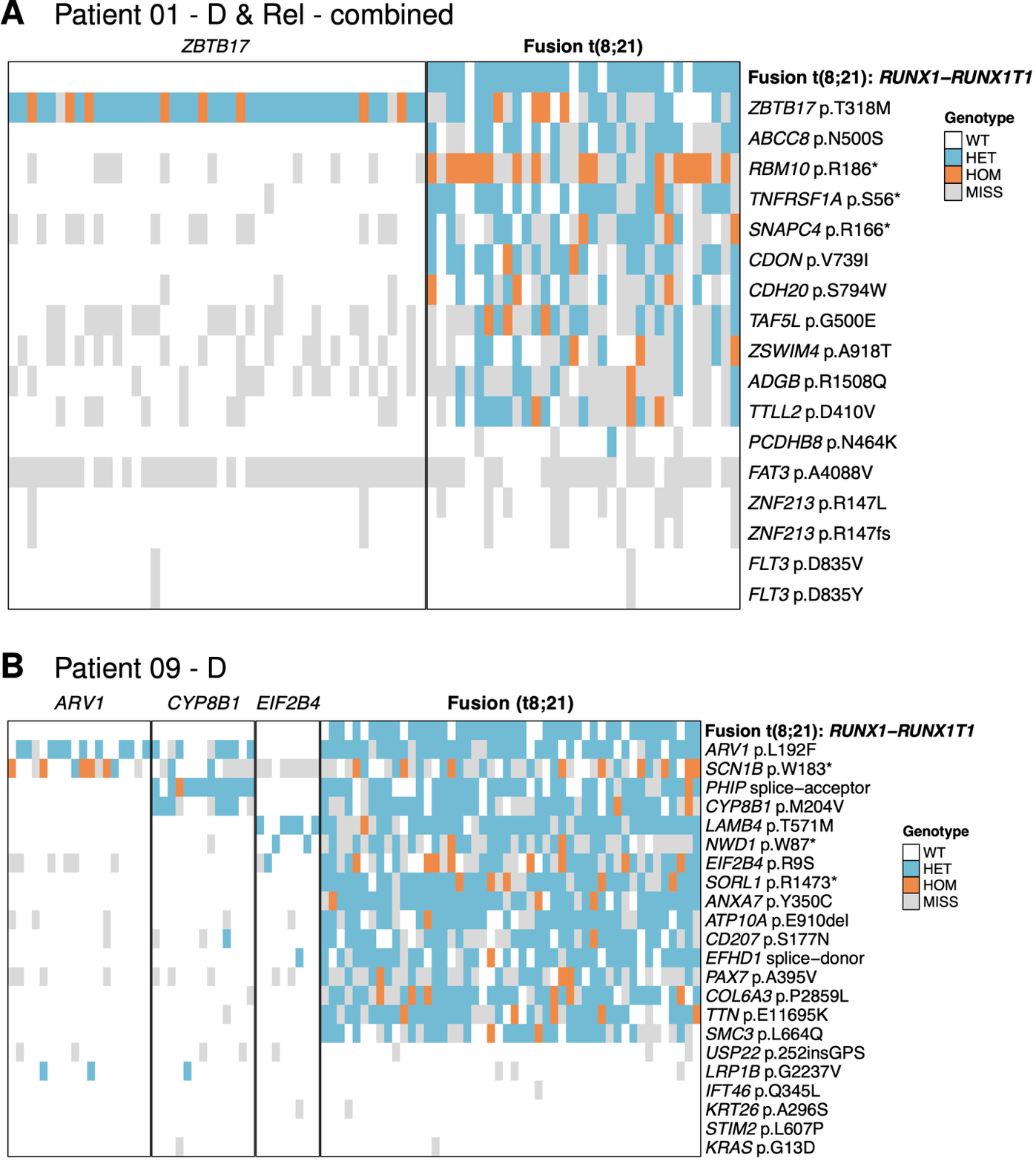
**

**Figure S22:** Violin plots showing percentage of mutated cells for each somatic variant in all patients based on the presence of fusion gene reads (blue: no fusion gene reads, orange: fusion gene reads are present) for diagnosis and relapse samples. P-values were calculated using Wilcoxon signed-rank test.

**
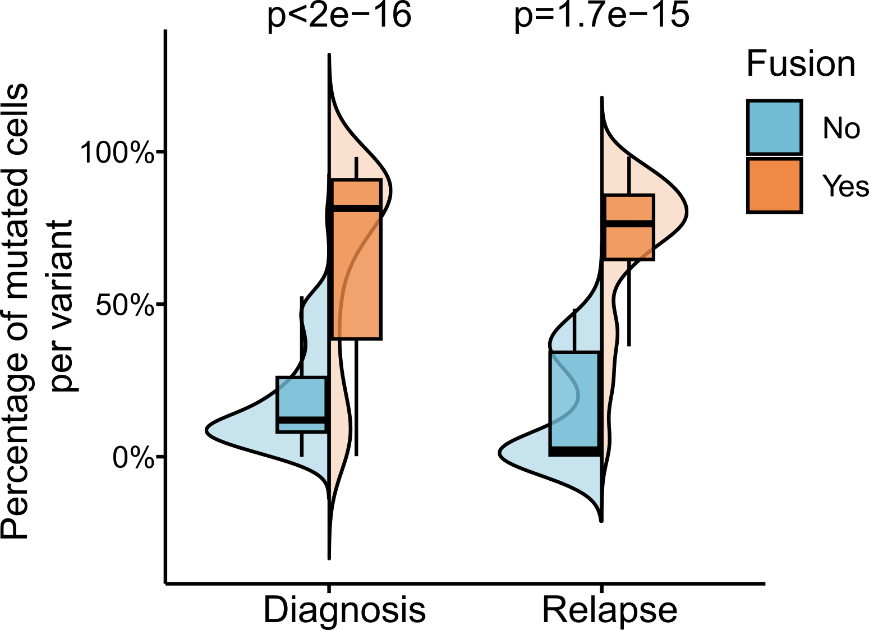
**

**Figure S23:** Bar plots showing the number of cells at complete remission (CR) grouped by the number mutated variants per cell for all patients with available CR samples. The total number of mutated cells are annotated in brackets. Numbers above the bars indicate the number of cells with the respective variant count.

**
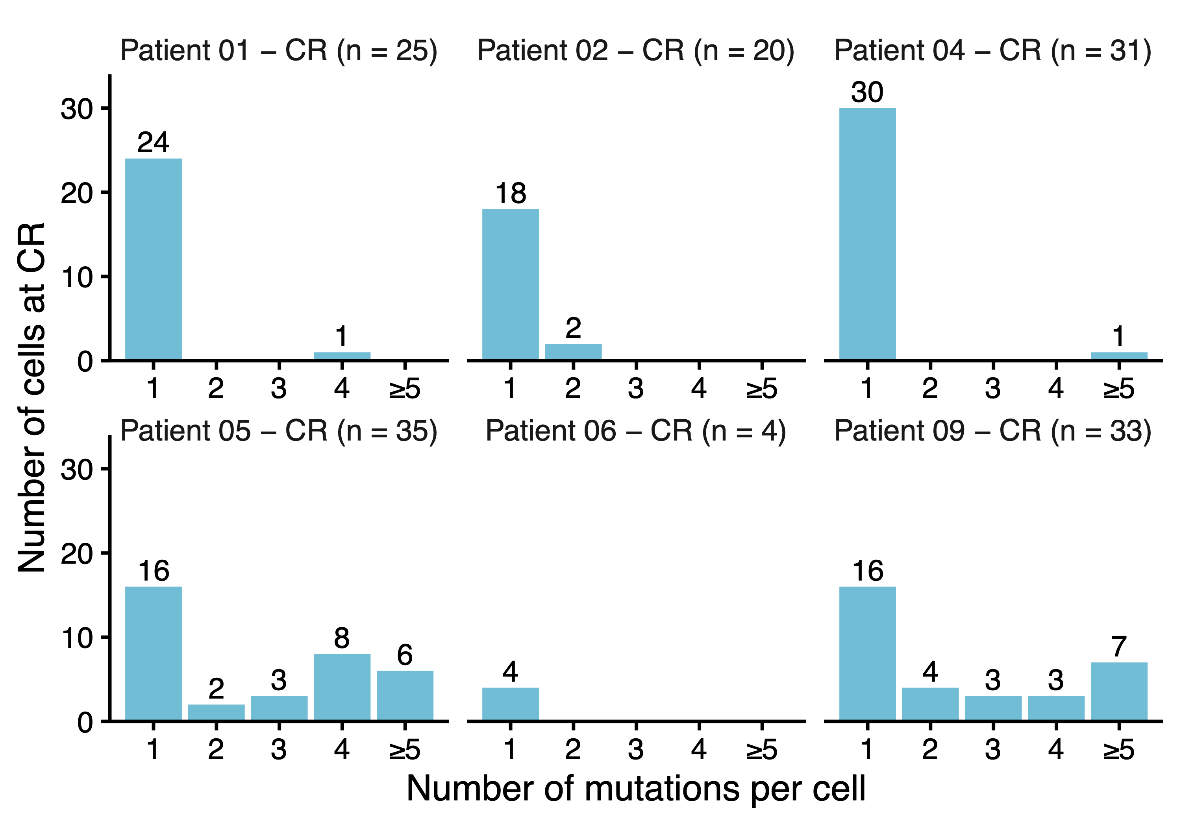
**

**Figure S24:** Heatmaps present mutated cells in columns and variants in rows at complete remission (CR) for patients **A** 01, **B** 02, **C** 04, and **D** 05. Variants annotated with a dot (•) are classified as mutated based on criteria including a depth ≥6 reads and a variant allele frequency (VAF) ≥20%. Cells are color-coded without thresholds: white represents a VAF of 0%; increasing VAFs with ≥1 alternative allele read are shown in a gradient from yellow to orange and grey indicates the loci is not covered. Number of cells with ≥1 variant mutated or gene fusion are provided in brackets.

**
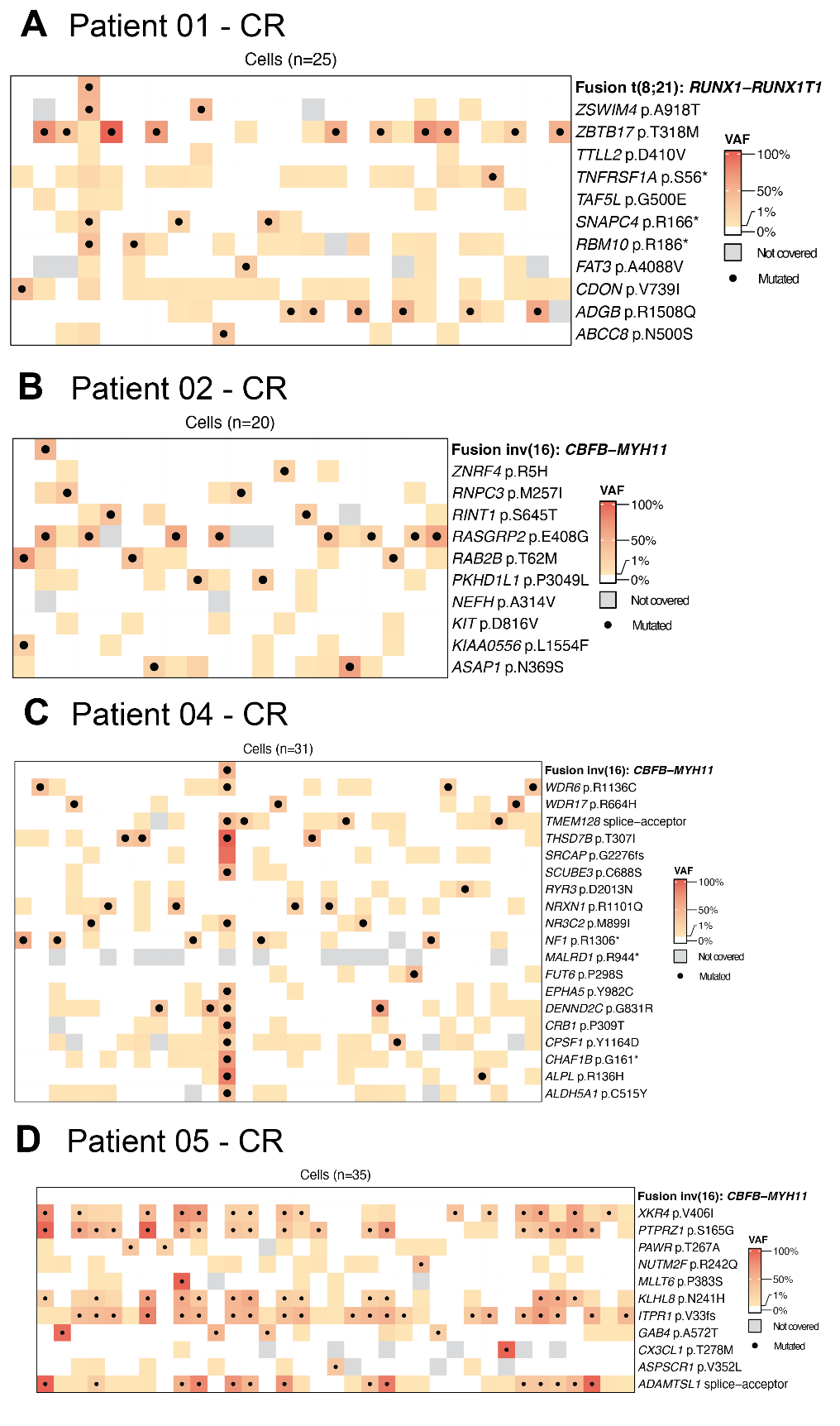
**

**Figure S25:** Heatmaps present mutated cells in columns and variants in rows at complete remission (CR) for patients **A** 06 and **B** 09. Variants annotated with a dot (•) are classified as mutated based on criteria including a depth ≥6 reads and a variant allele frequency (VAF) ≥20%. Cells are color-coded without thresholds: white represents a VAF of 0%; increasing VAFs with ≥1 alternative allele read are shown in a gradient from yellow to orange and grey indicates the loci is not covered. Number of cells with ≥1 variant or gene fusion are provided in brackets.

**
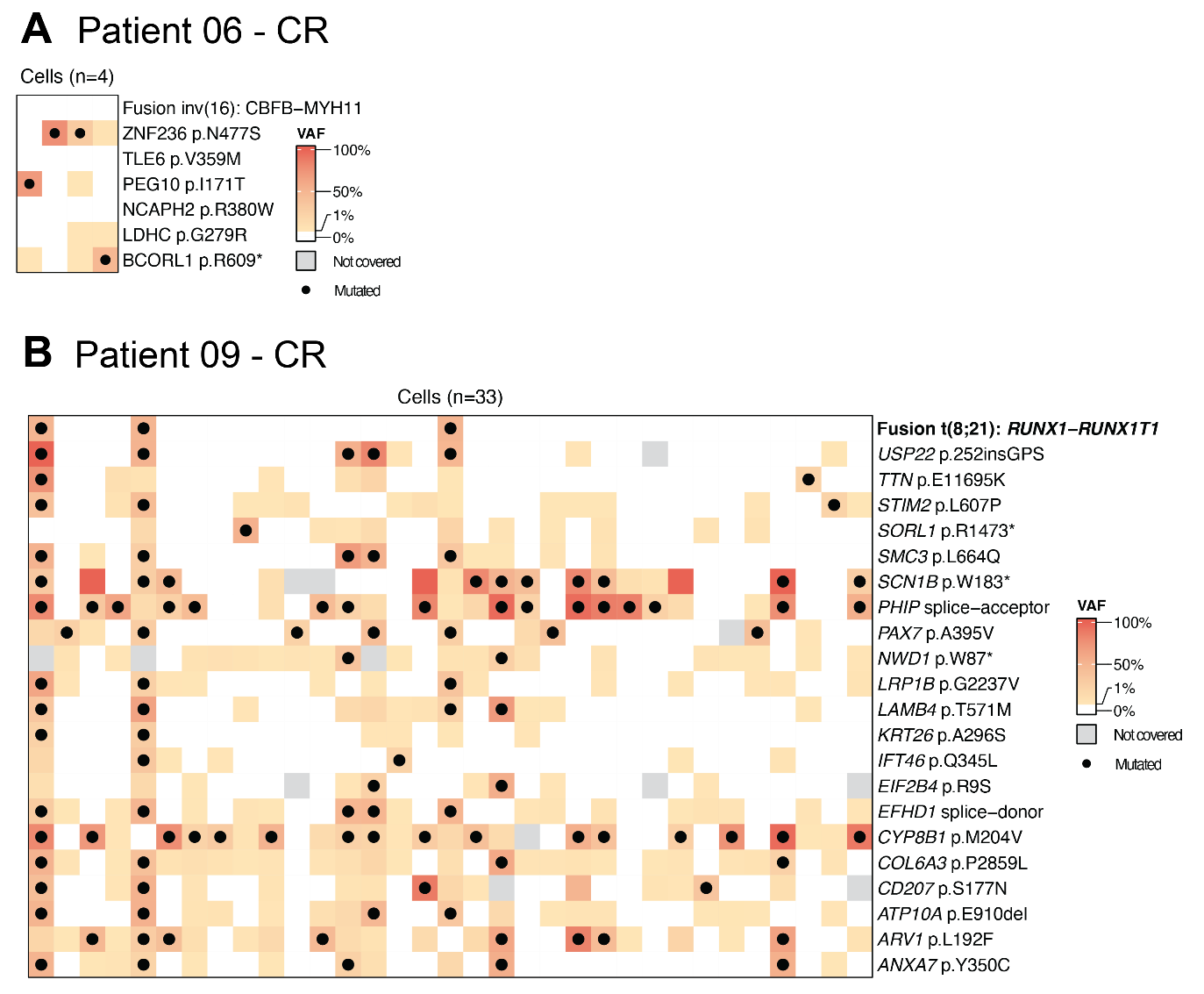
**

**References**

1. Mölder, F. *et al.* Sustainable data analysis with Snakemake. *F1000Res.* **10**, 33 (2021).

2. Arends, C.M. *et al.* Clonal hematopoiesis is associated with improved survival in patients with metastatic colorectal cancer from the FIRE-3 trial. *Blood* **139**, 1593-1597 (2022).

3. Arends, C.M. *et al.* Associations of clonal hematopoiesis with recurrent vascular events and death in patients with incident ischemic stroke. *Blood* **141**, 787-799 (2023).

4. Panagiota, V. *et al.* Clinical implications and dynamics of clonal hematopoiesis in anti-CD19 CAR T-cell treated patients. *HemaSphere* **7**, e957 (2023).

5. Arends, C.M. *et al.* Dynamics of clonal hematopoiesis under DNA-damaging treatment in patients with ovarian cancer. *Leukemia* **38**, 1378-1389 (2024).

6. Halik, A. *et al.* Genomic characterization of AML with aberrations of chromosome 7: a multinational cohort of 519 patients. *J Hematol Oncol* **17**, 70 (2024).

7. Bolger, A.M., Lohse, M. & Usadel, B. Trimmomatic: a flexible trimmer for Illumina sequence data. *Bioinformatics* **30**, 2114-2120 (2014).

8. Church, D.M. *et al.* Modernizing reference genome assemblies. *PLoS Biol.* **9**, e1001091 (2011).

9. Li, H. Aligning sequence reads, clone sequences and assembly contigs with BWA-MEM. *arXiv [q-bio.GN]* (2013).

10. Danecek, P. *et al.* Twelve years of SAMtools and BCFtools. *Gigascience* **10**(2021).

11. Broad, I. Picard Tools. *Broad Institute, GitHub repository, Accessed: 2020/08/20; version 2.20.0*.

12. Lai, Z. *et al.* VarDict: a novel and versatile variant caller for next-generation sequencing in cancer research. *Nucleic Acids Res.* **44**, e108 (2016).

13. Wang, K., Li, M. & Hakonarson, H. ANNOVAR: functional annotation of genetic variants from high-throughput sequencing data. *Nucleic Acids Res.* **38**, e164 (2010).

14. Pruitt, K.D., Tatusova, T. & Maglott, D.R. NCBI reference sequences (RefSeq): a curated non-redundant sequence database of genomes, transcripts and proteins. *Nucleic Acids Res.* **35**, D61-5 (2007).

15. Landrum, M.J. *et al.* ClinVar: improving access to variant interpretations and supporting evidence. *Nucleic Acids Res.* **46**, D1062-D1067 (2018).

16. Liu, X., Jian, X. & Boerwinkle, E. dbNSFP: a lightweight database of human nonsynonymous SNPs and their functional predictions. *Hum. Mutat.* **32**, 894-899 (2011).

17. Liu, X., Li, C., Mou, C., Dong, Y. & Tu, Y. dbNSFP v4: a comprehensive database of transcript-specific functional predictions and annotations for human nonsynonymous and splice-site SNVs. *Genome Med.* **12**, 103 (2020).

18. Chen, S. *et al.* A genomic mutational constraint map using variation in 76,156 human genomes. *Nature* **625**, 92-100 (2023).

19. Sherry, S.T. *et al.* dbSNP: the NCBI database of genetic variation. *Nucleic Acids Res.* **29**, 308-311 (2001).

20. Tate, J.G. *et al.* COSMIC: The Catalogue Of Somatic Mutations In Cancer. *Nucleic Acids Res.* **47**, D941-D947 (2019).

21. Ioannidis, N.M. *et al.* REVEL: An ensemble method for predicting the pathogenicity of rare missense variants. *Am. J. Hum. Genet.* **99**, 877-885 (2016).

22. Zhang, J. *et al.* The international cancer genome consortium data portal. *Nat. Biotechnol.* **37**, 367-369 (2019).

23. Caetano-Anolles, D. Fisher’s Exact Test.

24. Robinson, J.T. *et al.* Integrative genomics viewer. *Nat. Biotechnol.* **29**, 24-26 (2011).

25. Lee, S. *et al.* ITDetect: a method to detect internal tandem duplication of FMS-like tyrosine kinase (FLT3) from next-generation sequencing data with high sensitivity and clinical application. *BMC Bioinformatics* **24**, 62 (2023).

26. Watkins, T.B.K. *et al.* Pervasive chromosomal instability and karyotype order in tumour evolution. *Nature* (2020).

27. Watkins, T.B.K. *et al.* Refphase: Multi-sample phasing reveals haplotype-specific copy number heterogeneity. *PLoS Comput. Biol.* **19**, e1011379 (2023).

28. Van Loo, P. *et al.* Allele-specific copy number analysis of tumors. *Proc. Natl. Acad. Sci. U. S. A.* **107**, 16910-16915 (2010).

29. Wickham, H., François, R., Henry, L., Müller, K. & Vaughan, D. dplyr: A Grammar of Data Manipulation. (2023).

30. Wickham, H., Vaughan, D., Girlich, M. & Usher, K. Tidyr: Tidy messy data, V1. 2.0. (2022).

31. Hester, J. & Bryan, J. glue: Interpreted String Literals. (2022).

32. Bolker, B., Warnes, G.R. & Lumley, T. gtools: Various R Programming Tools. (2022).

33. Wickham, H., Hester, J. & Bryan, J. readr: Read Rectangular Text Data. (2023).

34. Fennell, T. & Homer, N. fgbio.

35. Genomes Project, C. *et al.* A global reference for human genetic variation. *Nature* **526**, 68-74 (2015).

36. Fu, Y., Mahmoud, M., Muraliraman, V.V., Sedlazeck, F.J. & Treangen, T.J. Vulcan: Improved long-read mapping and structural variant calling via dual-mode alignment. *Gigascience* **10**, giab063 (2021).

37. Stangl, C. *et al.* Partner independent fusion gene detection by multiplexed CRISPR-Cas9 enrichment and long read nanopore sequencing. *Nat. Commun.* **11**, 2861 (2020).

38. Sollier, E., Kuipers, J., Takahashi, K., Beerenwinkel, N. & Jahn, K. COMPASS: joint copy number and mutation phylogeny reconstruction from amplicon single-cell sequencing data. *Nat. Commun.* **14**, 4921 (2023).

39. Van Rossum, G. & Drake, F.L. *Python 3 Reference Manual*, (CreateSpace, Scotts Valley, CA, 2009).

40. Ushey, K., Allaire, J.J. & Tang, Y. reticulate: Interface to 'Python'. (2023).

41. Wickham, H. ggplot2: Elegant Graphics for Data Analysis. (2016).

42. Barrett, T. *et al.* data.table: Extension of `data.frame`. (2023).

43. Wickham, H. stringr: Simple, Consistent Wrappers for Common String Operations. (2022).

44. Kassambara, A. ggpubr: 'ggplot2' Based Publication Ready Plots. (2023).

45. Ooms, J. The jsonlite package: A practical and consistent mapping between JSON data and R objects. *arXiv [stat.CO]* (2014).

46. Wickham, H. & Bryan, J. readxl: Read Excel Files. (2023).

47. van den Brand, T. ggh4x: Hacks for 'ggplot2'. (2023).

48. Satas, G., Zaccaria, S., Mon, G. & Raphael, B.J. SCARLET: Single-cell tumor phylogeny inference with copy-number constrained mutation losses. *Cell Syst.* **10**, 323-332.e8 (2020).

49. Meschiari, S. latex2exp: Use LaTeX Expressions in Plots. (2023).

50. Morita, K. *et al.* Clonal evolution of acute myeloid leukemia revealed by high-throughput single-cell genomics. *Nat. Commun.* **11**, 5327 (2020).

51. Heuser, M. *et al.* 2021 Update on MRD in acute myeloid leukemia: a consensus document from the European LeukemiaNet MRD Working Party. *Blood* **138**, 2753-2767 (2021).

52. Sashittal, P., Zhang, H., Iacobuzio-Donahue, C.A. & Raphael, B.J. ConDoR: tumor phylogeny inference with a copy-number constrained mutation loss model. *Genome Biol.* **24**, 272 (2023).
